# DNA Condensates via Entanglement of String-like Structures Based on Anisotropic Nanotetrahedra

**DOI:** 10.1021/jacsau.5c00421

**Published:** 2025-06-10

**Authors:** Hong Xuan Chai, Kanta Kayanuma, Hiroaki Suzuki, Masahiro Takinoue

**Affiliations:** † Department of Life Science and Technology, 13290 Institute of Science Tokyo, 4259 Nagatsuta-cho, Yokohama, Kanagawa 226-8501, Japan; ‡ Department of Precision Mechanics, Graduate School of Science and Engineering, 12741 Chuo University, 1-13-27 Kasuga, Bunkyo-ku, Tokyo 112-8551, Japan; § Department of Computer Science, Institute of Science Tokyo, 4259 Nagatsuta-cho, Yokohama, Kanagawa 226-8501, Japan; ∥ Research Center for Autonomous Systems Materialogy (ASMat), Institute of Integrated Research, Institute of Science Tokyo, 4259 Nagatsuta-cho, Yokohama, Kanagawa 226-8501, Japan

**Keywords:** DNA condensate, DNA droplet, DNA hydrogel, DNA tetrahedron, Anisotropicity, Entanglement

## Abstract

Biomolecular condensates are attracting attention for their bioinspired functionalities and potential applications. However, the influence of biomolecular structural properties on the complex phase behaviors of biomolecular condensates remains poorly understood. In particular, the effect of component anisotropicity on condensates has been largely overlooked despite the existence of highly anisotropic biological condensates, such as heterochromatin. In this study, we report the formation of DNA condensates based on tetrahedron-shaped DNA nanostructures (Tetra-motifs). We designed an anisotropic Tetra-motif with two distinct pairs of sticky ends. Linkers corresponding to the stronger pair were introduced to form connections between Tetra-motifs. Unlike the flexible X-branched DNA nanostructures (X-motifs), the rigid and anisotropic structure of Tetra-motifs enabled their concatenation into extended, string-like structures. We found that these string-like structures of Tetra-motifs formed condensates even without cross-linking of multivalent motifs, relying solely on entanglement of these string-like structures. Mechanical and microfluidic experiments revealed that the resulting string-based condensates are highly deformable. Furthermore, we demonstrated the control of this string-based condensate by external stimuli, including UV irradiation and temperature changes, suggesting its potential as a stimuli-responsive material.

## Graphical abstract

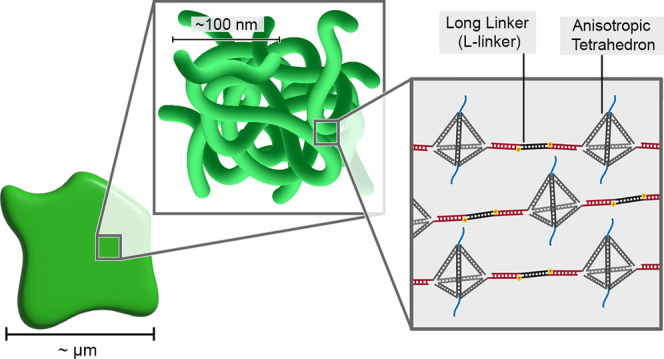

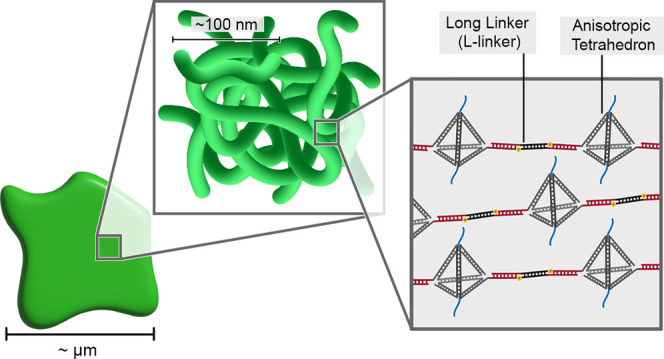

## Introduction

In recent years, there has been a lot of activity in constructing artificial cells,^1–9^ artificial organelles,^10–16^ and artificial nuclei^7,17–20^ inspired by living cells in the synthetic biology field. These artificial biological systems have used membrane vesicles, such as liposomes, that encapsulate biochemical reactions, such as protein expression systems,^21–24^ and recent studies often use phase-separated condensates of biopolymers, such as proteins and nucleic acids, and chemically synthesized polymers.^25–27^ The biomolecular condensates in living cells,^28,29^ such as nucleoli, P-bodies, and stress granules, are known to exert a variety of biological functions^30^ such as sensing, molecular localization, and activation/inactivation of molecular reactions inspired by such biological systems, and biomolecular condensates are attracting attention for their potential applications in drug delivery,^31–35^ material synthesis,^23,36–39^ molecular robotics,^40–43^ etc.

The biomolecular condensates are formed via multivalent associative interactions between biopolymers. For example, intrinsically disordered regions of proteins,^44^ nucleic acid base pairings,^45–48^ and charged biopolymers^15,49–54^ have been reported as essential associative interactions to form biomolecular condensates. Moreover, the flexibility of monomer structures is known to have an important role in phase separation behaviors,^55^ and the length of the multivalent branched arms affects the condensate growth rate.^56^ Therefore, although structural properties (entropic aspect) of biomolecules have been recognized to be very important in the formation of biomolecular condensates as well as the strength of intermolecular interactions (enthalpic aspect), the influence of most biomolecular structural properties on complex phase behaviors of biomolecular condensates remains to be unresolved.

DNA possesses excellent characteristics in programmability, base-pairing specificity, thermostability, and structural precision, just to name a few.^57^ DNA nanotechnology exploits the unique properties of DNA to create various nanostructures and nanodevices, enabling applications beyond its original biological functions, including medicine,^58,59^ computing,^2,60,61^ and material science.^62–64^ DNA programmability is also applied to sequence-designed synthesis of biomolecular condensates^27,46,65–67^ and understanding their phase behaviors.^55^ DNA condensates are formed through phase separation of multibranched DNA nanostructures^27,46,68^ or hybridization of long single-stranded DNAs.^65^ Their dynamic control has been well-studied,^69^ and their cell-mimicking properties, such as fission, fusion, and responsiveness to stimuli,^27,70^ make them suitable for dynamic tasks, including porous pattern formation,^71^ cargo capture,^27^ biosensing,^60^ logic gate operation,^60,61,72^ and locomotion.^73^ These condensates have a homogeneous inner structure and no anisotropicity, on average. Although there are some reports studying how anisotropic environments such as a surface affect the DNA condensate behaviors,^71,74^ anisotropicity of components consisting of condensates has not been investigated despite the fact that the biological condensate such as heterochromatins involves high anisotropicity contributed by chromatin string structures.

In this study, we report a DNA condensate composed of three-dimensional tetrahedron-shaped DNA nanostructures.^75^ The tetrahedron-shaped DNA nanostructures are expected to form string-like structures through the interconnection by DNA linkers because of their anisotropicity and rigidity (Figure 1). This property will contribute to investigating the effect of inner anisotropicity on the biomolecular condensate behaviors. As a result, we found that, unlike regular X-shaped DNA nanostructures, the anisotropic string-like structures composed of the tetrahedron-shaped DNA nanostructure could form phase-separated condensates even without multivalency of the monomers due to the entanglement of the string-like structures. In general, DNA polyhedra, including DNA tetrahedra,^75^ are known to be highly versatile for molecular encapsulation, as a DNA cage, enabling the functional control of the encapsulated molecule,^76,77^ and applicability to biomedical applications,^78–84^ the integration of the functionality of the DNA tetrahedron, and the potential for biomolecular condensation will promote the artificial biological system research as well as understanding the formation mechanisms of biomolecular condensates.

**Figure 1:**
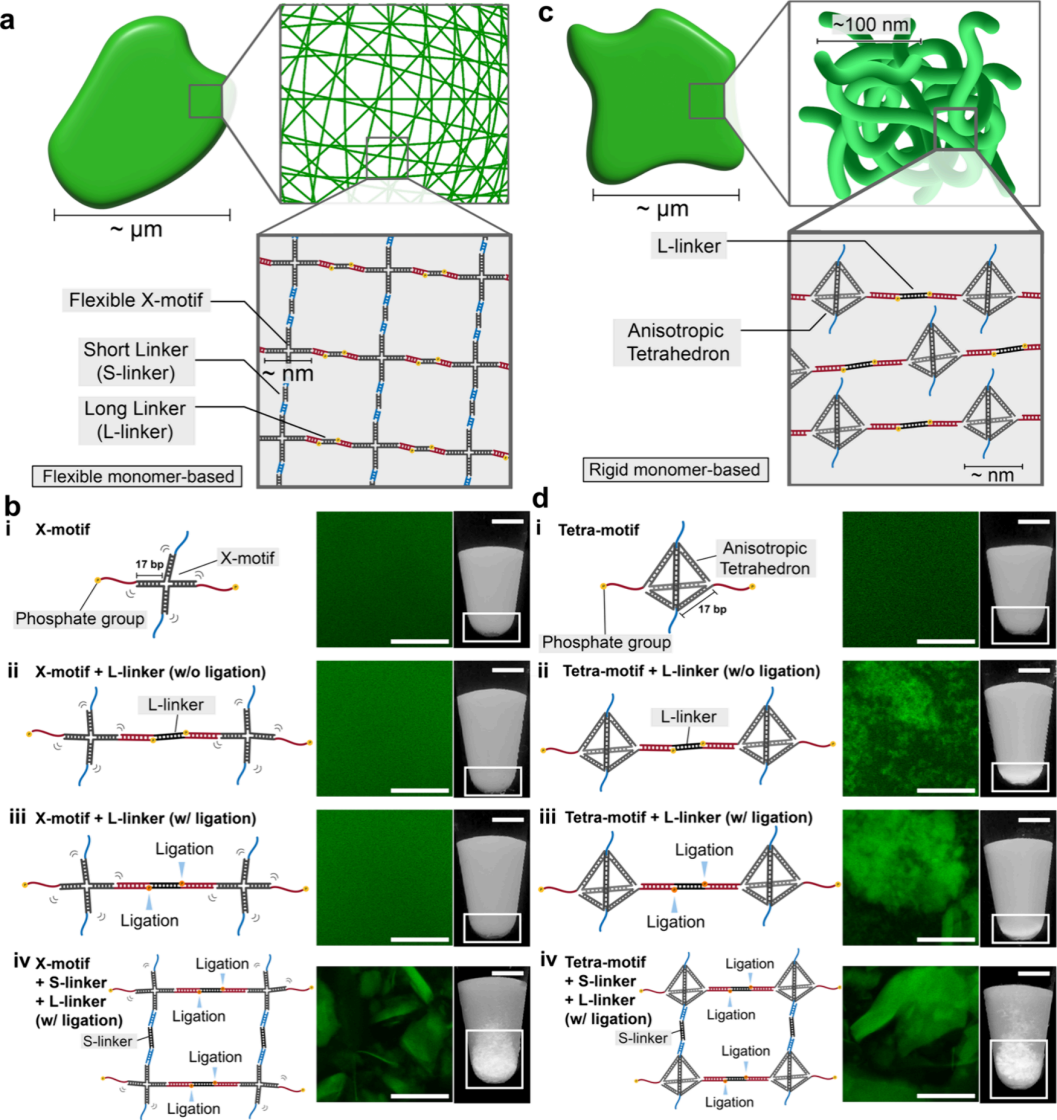
Construction of DNA condensates by two types of DNA nanostructures. (a) DNA condensates formed by X-branched DNA nanostructures (X-motifs) interacting with each other without a specific direction. X-motif is flexible, and its condensate is isotropic. (b) Illustration of X-motif under different conditions (left), corresponding confocal laser-scanning microscopy (CLSM) images (middle), and pictures of a pellet in a test tube (right): (i) X-motifs only, (ii) X-motifs + L-linkers (without (w/o) ligation), (iii) X-motifs + L-linkers (with (w/) ligation), and (iv) X-motifs + L-linkers + S-linkers (w/ligation). L-linker: a long-sticky-end DNA linker. S-linker: a short-sticky-end DNA linker. (c) Illustration of DNA condensate consists of entangled string-like structures made up of anisotropic tetrahedron-shaped DNA nanostructures (Tetra-motifs) concatenated by the L-linkers. (d) Illustration of anisotropic Tetra-motif under different conditions (left), corresponding CLSM images (middle), and pictures of a pellet in a test tube (right): (i) Tetra-motifs only, (ii) Tetra-motifs + L-linkers (w/o ligation), (iii) Tetra-motifs + L-linkers (w/ligation), and (iv) Tetra-motifs + L-linkers + S-Linkers (w/ligation). Scale bars for the CLSM images: 50 μm. Scale bars for the pellets: 2 mm.

## Results and Discussion

First, we used an X-branched DNA nanostructure (X-motif) to form the previously reported DNA condensates^27,46,85,86^ (Figure 1a). The X-motif has two types of sticky ends at the end of the branched arms: two long sticky ends for more stable hybridization and two short sticky ends for less stable hybridization. The X-motifs can be connected with long-sticky-end DNA linkers (L-linkers) and short-sticky-end DNA linkers (S-linkers) to form DNA condensates. Since the branch center angle of the X-motif is not fixed, the X-motif is flexible; thus, the X-motif condensate is isotropic on average due to flexible structural changes of the X-motif, despite the stability difference in the sticky ends. However, we note that the actual flexibility of the X-motif can depend on both sequence and ionic condition. Even with two unpaired thymidines at the junction, a stiffer conformation may form due to a X-stacking between adjacent arm, especially under high salt concentrations.^87^ Figure 1b shows the experiments to form DNA condensates of the X-motif under four different conditions. No DNA condensates were formed under conditions without L-linkers or S-linkers (Figure 1b­(i)), with L-linkers without ligation (Figure 1b­(ii)), and with L-linkers with ligation (Figure 1b­(iii)). DNA condensates were formed only when both L- and S-linkers were present (Figure 1b­(iv)). These results were consistent with those of previous studies.^27^

Next, we designed a tetrahedron-shaped DNA nanostructure (Tetra-motif) that has two long and two short sticky ends at the vertices of the tetrahedron. Among the various DNA polyhedra reported,^75,88^ the DNA tetrahedron is well-known for its ease of assembly, simplicity of design,^89^ and mechanical robustness.^75^ To ensure motif stability comparable to that of the X-motif, the edge length of the Tetra-motif was set to 17 bp. The connections between Tetra-motifs via linkers generate DNA condensates (Figure 1c). The Tetra-motif is rigid, unlike the X-motif because a pair of Tetra-motif edges in the skew position is perpendicularly fixed by the other edges. To be more precise, the edge created by two vertices for the two long sticky ends and the other for the two short sticky ends are perpendicular; i.e., the Tetra-motif is an anisotropic structure. Thus, when Tetra-motifs are connected through the L-linkers without S-linkers, concatenated Tetra-motifs are expected to form highly anisotropic string-like structures due to the Tetra-motif’s anisotropicity and rigidity, inspired by chromatin-like or nucleoid-like string structures. Similar linear assembly of DNA tetrahedra have indeed been demonstrated previously,^90^ supporting feasibility of this design. On the other hand, the S-linker can contribute to the modulation of the condensate physical properties through the addition of the multivalent interactions between string-like structures. Notably, more visible material was observed with both L- and S-linkers (Figure 1d­(iv)), where a fully cross-linked structure is expected compared with the smaller amount formed with L-linkers alone.

Figure 1d shows the experiments to form DNA condensates of the Tetra-motifs under four different conditions. In the case of the Tetra-motif, no DNA condensates formed in the condition without linkers (Figure 1d­(i)), such as the X-motifs. However, interestingly, DNA condensates were formed just by including the L-linkers even without the S-linkers, regardless of whether the linkers were not ligated (Figure 1d­(ii)) or not ligated (Figure 1d­(iii)). Ligation introduced in Figure 1b­(iii) and Figure 1d­(iii) was intended to strengthen the concatenation between motifs for a more extended structure. In the case of the X-motif, these conditions did not result in the formation of DNA condensates (Figure 1b­(ii) and (iii)). In addition, DNA condensates were formed upon the addition of the S-linker (Figure 1d­(iv)), like X-motif (Figure 1b­(iv)), which agree with the previously reported DNA tetrahedron-based cross-linked hydrogel.^91,92^ The formation of DNA condensates using Tetra-motifs with only long sticky ends and without short sticky ends (Tetra-motif_noShortSE) was also confirmed and showed the same results (Figure S1).

The Tetra-motifs can be concatenated by the L-linkers to form a more rigid string-like structure due to the Tetra-motif’s anisotropicity and rigidity, unlike the X-motifs. The formation of a string-like structure may result in a difference in the formation of condensates between the Tetra-motif and the X-motif. In addition, the bulk geometry of the Tetra-motif may favor linear, string-like assembly due to steric hindrance, which restricts bending and folding between adjacent motifs. It is hypothesized that ‘entanglement’ of the rigid string-like structure formed by the Tetra-motifs contributes to the formation of the Tetra-motif condensates in the case of the lack of the S-linkers, i.e., without interconnections between the string-like structures. To understand the nontrivial formation mechanism of the Tetra-motif condensate, we investigated the nanometer- and micrometer-scale physical and structural properties of the Tetra-motif concatenation and its condensates below.

Physical entanglement is a phenomenon involving the intertwining of polymer chains, resulting in topological constraints on their motion.^93,94^ Unlike cross-linked DNA hydrogels, whose molecular motion is primarily restricted, physically entangled polymers enable partial mobility by allowing the polymer chains to slide against each other.^93^ This dynamic behavior enables structural rearrangement without disrupting cross-linking.

To verify the specificity of the L-linker, three different variants of Tetra-motif were used (Figure 2a): the original Tetra-motif described above, a DNA tetrahedron with two long and two short sticky ends; Tetra-motif_noShortSE, with only two long sticky ends and without short sticky ends, to confirm that the L-linkers interact with long sticky ends as designed; and Tetra-motif_noSE, without any sticky ends, to ensure that the L-linkers do not unexpectedly interact with the tetrahedral body. Figure 2a shows a native polyacrylamide gel electrophoresis (PAGE) result for Tetra-motif variants, the L-linker, and their mixtures. The presence of aggregate stuck in the wells (Figure 2a­(i), (ii), (iv), and (v); enclosed by squares) indicates the formation of Tetra-motif variant condensates via interaction between the L-linker and the Tetra-motif variants. The L-linker was found to interact only with the original Tetra-motif (Figure 2a­(ii) and (v)) and Tetra-motif_noShortSE (Figure 2a­(i) and (iv)) but not with Tetra-motif_noSE (Figure 2a­(iii)), confirming the specificity of the L-linker connection. Additionally, the ligation between the Tetra-motifs and the L-linker, designed to enhance the interaction between the two Tetra-motifs, was verified in the presence of the ligation product with an expected length of 120 nt in the denaturing PAGE result (Figure 2b). These results demonstrate that the interaction between the Tetra-motifs and the L-linkers occurred as designed. Additional data, including the case of X-motif, are available in Figure S2.

**Figure 2:**
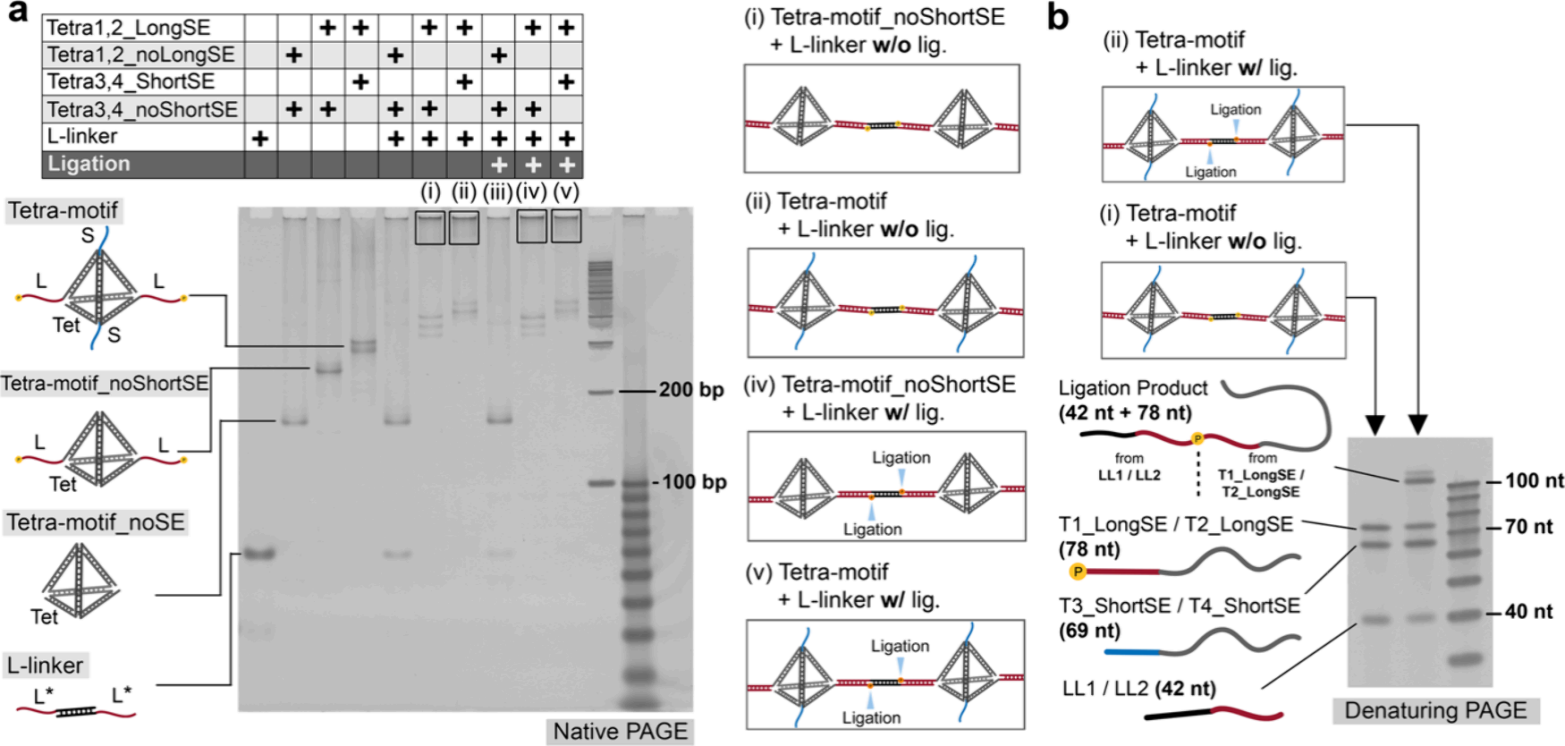
Verification of Tetra-motif assemblies using gel electrophoresis. (a) Native PAGE analysis for Tetra-motif variants (original Tetra-motif, Tetra-motif_noShortSE, and Tetra-motif_noSE), L-linker, and their mixtures. (b) Denaturing PAGE analysis for the component single-stranded DNA in the original Tetra-motifs and the L-linkers after the ligation.

The atomic force microscopy (AFM) was used to investigate the nanometer-scale structures in the Tetra-motif condensates and the X-motif condensates (Figure 3). To clearly visualize the internal nanostructures comprised in the DNA condensates, we spin-coated a solution containing DNA condensates onto a freshly cleaved mica sheet to spread them thinly. The “arm” part of the solution spread out thinly enough and was cut out and then treated with NiCl_2_ to fix DNA nanostructures on the mica surface, followed by the AFM observation (Figure 3a).

**Figure 3:**
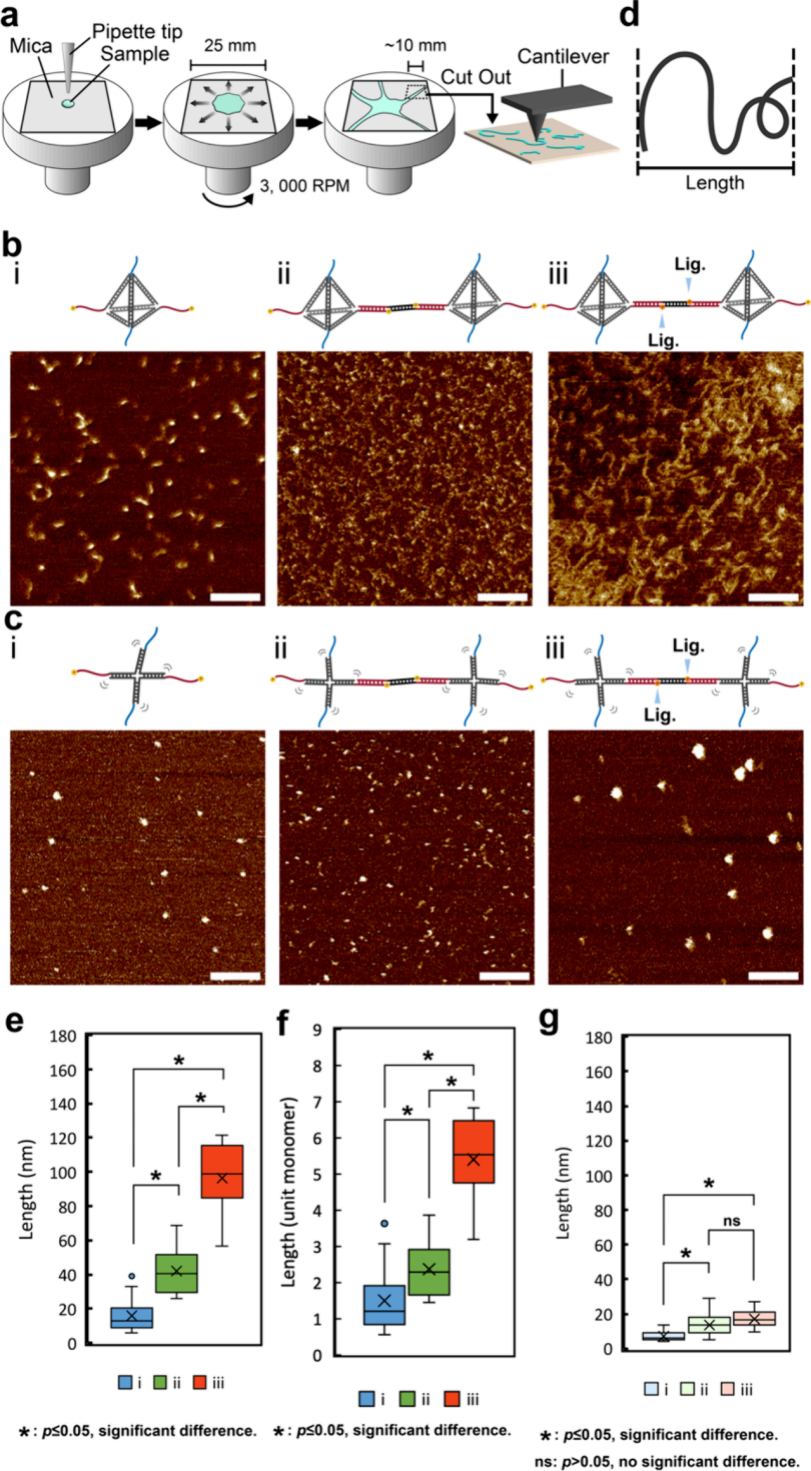
Atomic force microscopy (AFM) analysis of nanostructures composing condensates. (a) Illustration depicting sample preparation through the spin coating. (b) Illustration (top) and representative AFM images (bottom) for Tetra-motifs under three conditions: (i) Tetra-motifs only, (ii) Tetra-motifs + L-linkers (w/o ligation), and (iii) Tetra-motifs + L-linkers (w/ligation). (c) Illustration (top) and representative AFM images (bottom) for X-motifs under three conditions: (i) X-motifs only, (ii) X-motifs + L-linkers (w/o ligation), and (iii) X-motifs + L-linkers (w/ligation). (d) Schematics depicting the method to measure apparent end-to-end string lengths. (e) Box plot of mean string lengths (in nanometers, nm) from three independent replicates per condition, with 20 structures measured per replicate. Data correspond to panel (b). (f) Box plot of estimated string lengths expressed in number of monomer units. The unit length per Tetra-motif was estimated to be 10.7 nm based on direct measurements. For Tetra-motifs with L-linkers, the estimated unit length was 17.9 nm, assuming 1 base pair = 0.34 nm. Data correspond to panel (b). (g) Box plot of mean string lengths (in nanometers, nm) from three independent replicates per condition, with 20 structures measured per replicate. Data correspond to panel (c). Box plots indicate the median (center line), interquartile range (box), and 1.5× IQR (whiskers). The “X” marks the group mean. Statistical comparisons were performed using two-tailed Welch’s *t*-tests. Significance is indicated as *p* ≤ 0.05 (*) and *p* > 0.05 (ns). Scale bars represent 100 nm. Additional data are shown in Figure S3.

Figure 3b shows the AFM images for the Tetra-motif only (Figure 3b­(i)), Tetra-motif with L-linkers without ligation (Figure 3b­(ii)), and that with ligation (Figure 3b­(iii)). In Figures 3b­(i) and (ii), many dot-like or small connected structures were observed. In contrast, many obvious rigid string-like nanostructures were observed in Figure 3b­(iii); these structures are what we expected when designed. This AFM image directly demonstrates that the Tetra-motif “strings” were formed in the condensates after connecting Tetra-motifs and L-linkers ligation. The fact that the condensates were unraveled into many strings by the shear stress of the spin coating shows that the strings were not cross-linked because of the lack of the S-linkers. Figures 3c­(i)–(iii) show that individual particles with slight variations in size were formed across the three conditions in the case of the X-motif. The larger particles in Figure 3c­(iii) might suggest the formation of folded structures of very flexible strings. Although only a few large random aggregates were observed even in Figure 3c­(i), the addition of L-linkers in Figure 3c­(ii) resulted in more extended structures that were more statistically dominant (see also Figures S3–S6), leading to a greater average end-to-end length as shown in Figure 3g. Anyway, there is no clear evidence of string-like structure formation in the case of the X-motif, even in the presence of L-linkers.

To further understand this observation, the apparent end-to-end lengths of the structures were measured (Figure 3d) and are presented by box plots (Figures 3e, 3f, and 3g). For the Tetra-motif (Figure 3e), the length of the string-like structure increased significantly with the addition of the L-linker and further increased significantly with ligation. Next, we converted the length of the string-like structures into the number of unit monomers (Figure 3f), where the unit monomer is one Tetra-motif only for Figure 3b­(i), and the connected structure of one Tetra-motif and one L-linker for Figures 3b­(ii) and (iii). The addition of the L-linker to the Tetra-motif resulted in only a slight increase in the length to approximately two units of the monomer. This result suggests dimeric and trimeric compositions of the condensate. With ligation, the length of the string-like structure increased significantly to approximately 5–6 units of the monomers, suggesting the significance of ligation in strengthening the L-linker connection between Tetra-motifs. For the X-motif, the absence of string-like structures was supported by only a slight increase in the length of the measured structures (Figure 3g). This is likely because the formation of a string-like structure is entropically unfavorable, largely because of the structural flexibility of the X-motif,^55^ which supports our observations in Figure 1. The AFM images also demonstrated that the string-like structures formed by connected DNA motifs in the Tetra-motif samples were longer than those observed in the X-motif samples, despite the smaller size of the Tetra-motif. This suggests that the connection efficiency of the Tetra-motif was higher, likely due to its structural rigidity. In contrast, the greater flexibility of the X-motif may have reduced its effective connectivity, consistent with the absence of string-like structure formation observed. The AFM imaging enabled the direct observation of string-like structures composed of interconnecting Tetra-motif via L-linkers and suggested the effectiveness of the strategy to increase the length of string-like structures with ligation.

A macroscopic investigation was conducted by introducing mechanical stress via pipet tip manipulation (Figure 4a). The condensates were drawn slightly into the pipet tip and gently pulled away for stretching. We found that the condensates without cross-linking by S-linkers can be stretched into fibrous structures without fragmentation (Figures 4a­(i) and (ii); Movies S1 and S2), suggesting that the string-like structures composing the condensates were rearranged into more parallel orientation. In contrast, the condensate cross-linked by S-linkers exhibited a gel-like morphology (Figure 4a­(iii)), which remained unchanged, with no fibrous structure observed after manipulation (Movie S3). These observations suggest that the condensates without cross-linking by S-linkers (Figures 4a­(i) and (ii)) were formed via entanglement of the string-like structures, unlike the cross-linked condensate (Figure 4a­(iii)). Hereafter, the condensates without/with ligation and without cross-linking by S-linkers (Figures 4a­(i) and (ii)) are named nonligated/ligated string-based condensates; the condensates with both ligation and cross-linking by S-linkers (Figure 4a­(iii)) are named cross-linked condensate.

**Figure 4:**
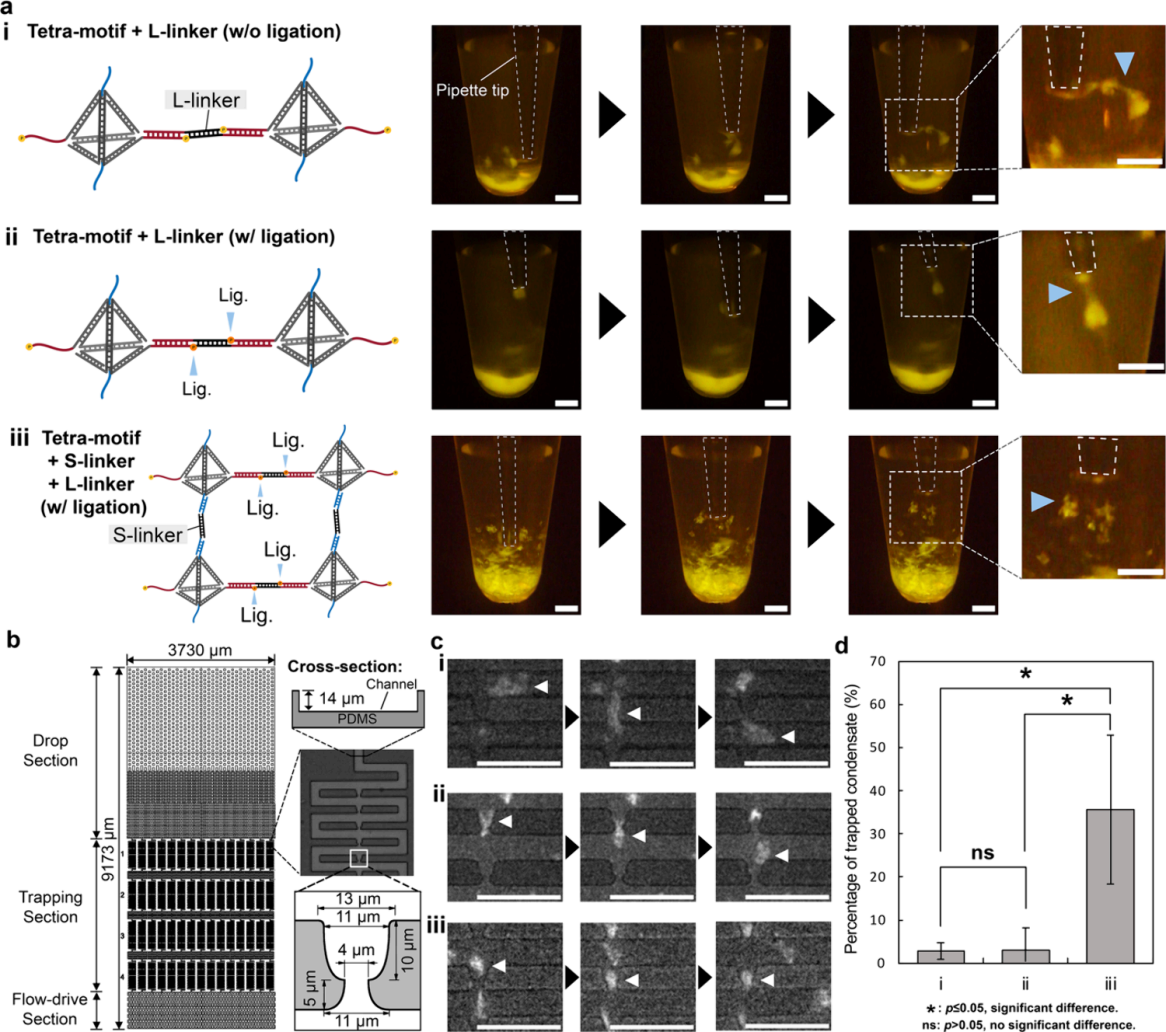
Investigation of condensate physical properties across three conditions: (i) Tetra-motif + L-linker (w/o ligation), (ii) Tetra-motif + L-linker (w/ligation), and (iii) Tetra-motif + L-linker + S-linker (w/ligation). (a) Illustration (left) and images of condensates manipulated with a pipet tip (indicated by blue arrows) in a test tube (right), demonstrating physical properties under the three conditions. Pipette tips are labeled with dotted lines. Points of interest are indicated by blue triangles. Scale bars: 1 mm. See Movies S1, S2, and S3 for details. The condensate (i) is named nonligated string-based condensates; (ii) is named ligated string-based condensates; and (iii) is named cross-linked condensates. (b) Schematic of the microfluidic trap device used to quantify condensate particle trapping under the same conditions. (c) Representative behavior of condensates under each condition, showing whether they squeeze through or become trapped in the device. Scale bars: 50 μm. See Movies S4, S5, and S6 for details. (d) Bar chart showing the percentage of trapped condensates for conditions (i), (ii), and (iii). One-tailed Welch’s *t* test was used for statistical analysis. Significance is indicated as *p* ≤ 0.05 (*), and *p* > 0.05 (ns). Size-frequency distribution of condensate particles pass-through and trapped are shown in Figure S8.

To further study the morphological changes of the condensate in response to the shear, an open microfluidic device with deterministic hydrodynamic traps^95^ was adopted. In this device, the hydrophilic treatment was applied only inside the open microchannel to induce spontaneous capillary flow. In practice, one microliter of diluted samples of condensates was aliquoted onto the drop section, and then the particles of condensates were allowed to flow spontaneously through the channel with traps designed to trap 10 μm particles (Figure 4b and Figure S7). The behavior of condensate particles flowing in the channel, typically at a flow velocity of (∼5 mm/s, was observed particularly at the trap (Figure 4c). For both nonligated and ligated string-based condensates, most particles could deform and squeeze through the narrow section of the trap (Figures 4c­(i) and (ii)) by temporarily conforming their shape onto the trap and recovered after passing through (Movies S4 and S5). However, a considerable portion of the particles of the cross-linked condensate could not squeeze through the trap (Figure 4c (iii); Movie S6). To further quantify this behavior, the flow of condensates in three parallel channels (Movie S7, S8, and S9) was captured. By enumerating the number of trapped particles in the condensates, we found that approximately 35% of the particles of the cross-linked condensate could not pass through the trap, which is significantly higher than that of the other two string-based condensates (Figure 4d). The string-based condensates were found to be more flexible and adaptable than cross-linked condensates. Importantly, since particles of similar size were observed across all three conditions (Figure S8), the increased trapping of the cross-linked condensates is unlikely to be due to size effect alone but primarily attributed to their reduced flexibility.

Fluorescence recovery after a photobleaching (FRAP) experiment was performed to further investigate the molecular dynamics of the condensates (Figure 5). An area of 6 μm diameter was bleached and appeared as a circular dark spot at *t* = 0 min in Figure 5a. The fluorescence intensity at the same spot was measured again 5 min after photobleaching. This single time point (*t* = 5 min) was selected to ensure a sufficiently high signal-to-noise ratio while minimizing sample drift and photobleaching effects, which could interfere with recovery measurements in slowly recovering samples. At *t* = 5 min, the dark spot almost disappeared for a Tetra-motif solution (Figure 5a­(i)) and faded for a nonligated string-based condensate (Figure 5a­(ii)). For a ligated string-based condensate (Figure 5a­(iii)), no significant change was observed 5 min after photobleaching. The quantified data are plotted as shown in Figure 5b, reflecting our observation in Figure 5a. The ligated string-based condensate recovered its fluorescence intensity with normalized intensity at less than 5% of that before photobleaching, which is much lower than the nonligated string-based condensate. This result suggests that ligation increased the viscosity of the string-based condensates.

**Figure 5:**
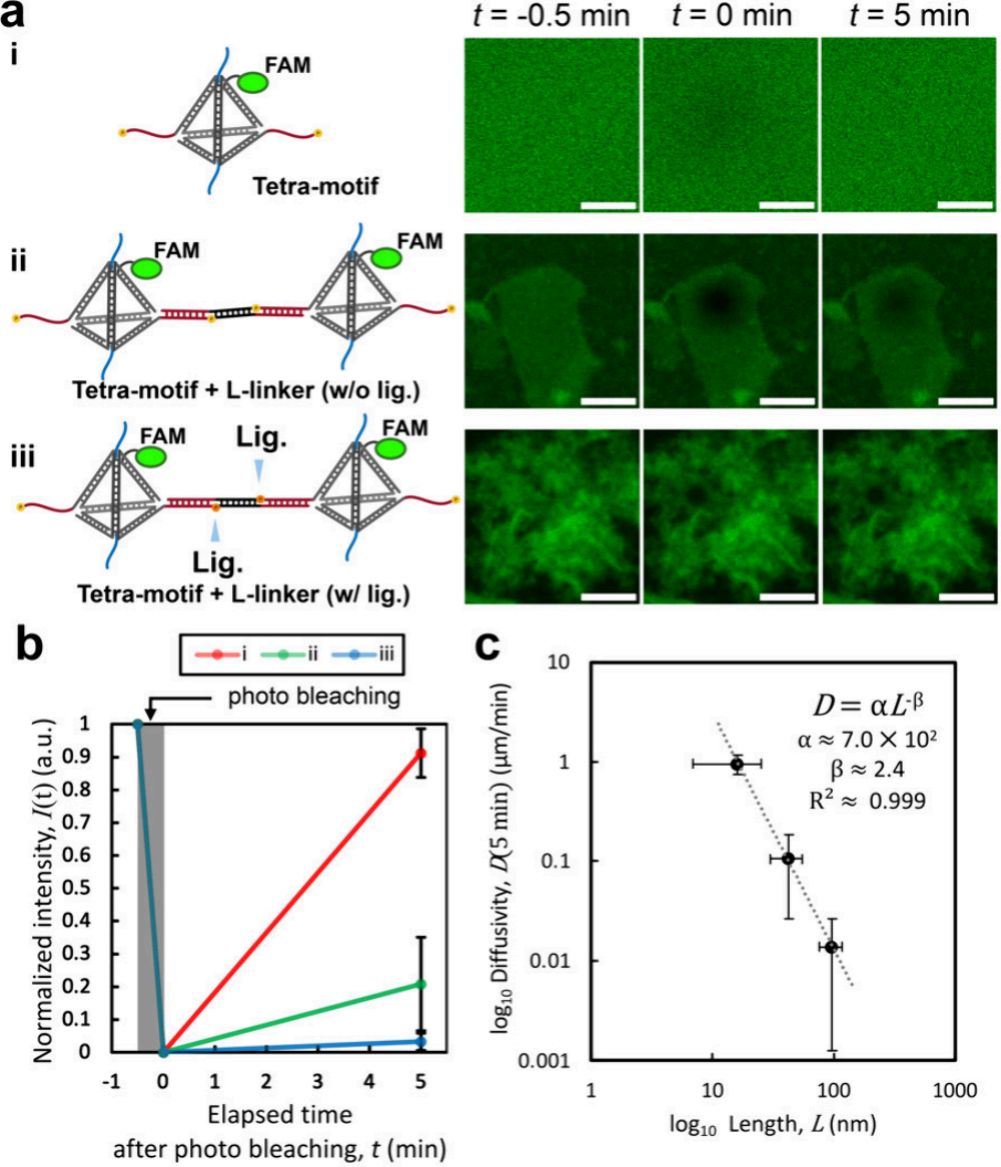
Analysis of the molecular dynamics using FRAP. (a) Illustration with the location of FAM labeling indicated (left) and representative images at prebleaching, immediately postbleaching, and 5 min postbleaching (right) under three different conditions: (i) Tetra-motif, (ii) Tetra-motif + L-linker (w/o ligation), and (iii) Tetra-motif + L-linker (w/ligation). Scale bars: 20 μm. (b) Plot of fluorescence recovery highlighting differences in recovery levels, corresponding to the same conditions (i), (ii), and (iii) as shown in (a). Data analysis is explained in Figure S9. (c) Log–log plot of mean diffusivity against the mean length of the string-like structure of the condensates. Error bars represents ± standard deviation (SD) of *D* values calculated from normalized intensity at 5 min postbleaching. Details in Figure S10.

The fluorescence intensity time variation is described as *I*(*t*) = *I*_∞_[1 – exp­(−*t*/τ) ],^96^ where *I*_∞_ represents the equilibrium fluorescence intensity after complete recovery; *t* is the elapsed time after photobleaching; and *τ = w* ^2^/(4*D*) is the characteristic recovery time constant, with *w* as the radius of bleached spot and *D* as the diffusion coefficient or diffusivity of the DNA condensate. In our analysis, fluorescence intensity was normalized to the prebleach level. Assuming full recovery, we approximated *I*_∞_ ≈ *I*_pre_. *D* was determined by fitting the FRAP recovery equation, ln­(1 – *I*(*t*)/*I*_∞_) = −(4*D*/*w* ^2^)·*t*, to the intensity data (Figures 5b and S7).

By combining the data from Figure 3 and the calculated *D*, we obtained a plot (Figure 5c) of *D* against the length of the string-like structure of the condensate (*L*) in Figure 3e. The data show a relationship of *D* ∝ *L* ^–2.4^ (Figure 5c). By assuming the reptation model^93^ for polymers, the diffusivity in condensates (*D*_*rep*_) is predicted to be *D*_*rep*_ ∝ *L* ^–2^, where *N* represents the number of monomer. Assuming that the polymer length *L* is proportional to *N*, it follows that *D*_*rep*_ ∝ *L* ^–2^.

The slight deviation from the theoretical *L* ^–2^ value was likely due to experimental variability in the measured string-like structure lengths and the heterogeneity in the entanglement of the condensates due to strings length variation. We also assumed full fluorescence recovery (*I*_∞_ ≈ *I*_pre_) for normalization, in which any deviation could affect the estimation of *D*, particularly for slowly recovering samples. Nonetheless, the observed trend of increasing length of the string-like structure through ligation with increasing viscosity of the DNA condensate agrees with the reptation model and further supports the interpretation that the DNA condensate is an entangled polymer system.

Finally, we demonstrate control of the string-based condensates by external stimuli. Figures 6a and 6b show the photocontrol of the L-linker parts. Photocleavable (PC) spacers were inserted into the L-linkers of the ligated string-based condensate (Figure 6a). The PC spacer insertion allows the intermonomer connection to be cleaved upon ultraviolet (UV) irradiation. Three different conditions were prepared for investigation: ligated string-based DNA condensates without the PC spacers but with UV irradiation (Figure 6b­(i)), with the PC spacers but without UV irradiation (Figure 6b­(ii)), and with the PC spacers and UV irradiation (Figure 6b­(iii)). As shown in Figure 6b, only the DNA condensates formed with the PC spacer-inserted L-linkers (Figure 6b­(iv)) disintegrated after 3 min of UV irradiation. This observation suggests the excision of intermonomer connections by UV irradiation, breaking down the string-like structures or polymers into individual monomers that are freely dispersed. This not only demonstrates the photoresponsivity of the string-based condensates but also strongly affirms the essential role of the string-like structures in condensate formation.

**Figure 6:**
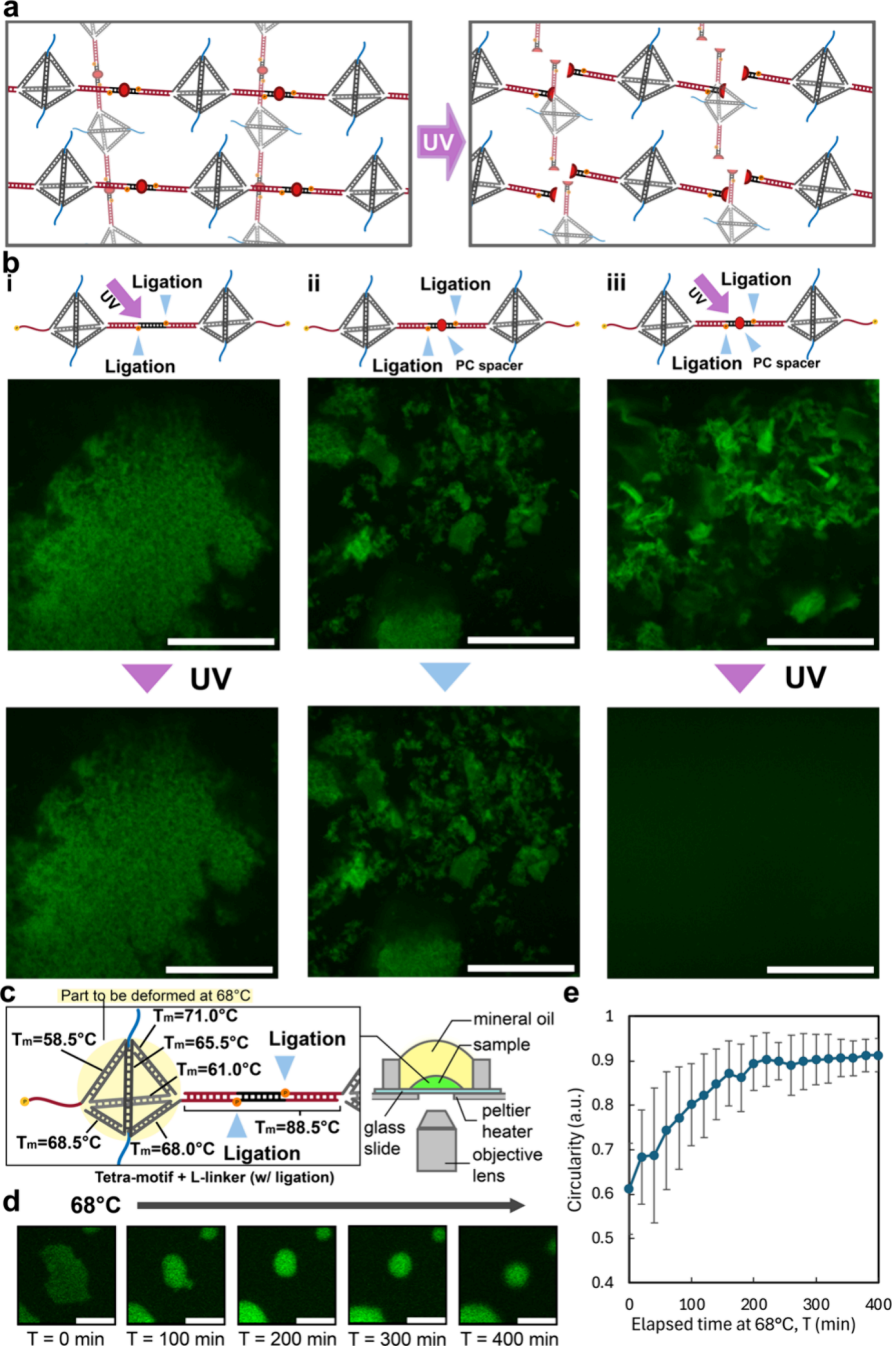
External stimuli control of ligated string-based condensates. (a) Illustration depicting experimental design investigating disintegration of DNA condensate formed by string-like structure connected via photocleavable (PC) spacer-inserted L-linker, following ultraviolet (UV) irradiation. (b) Illustration (top) and representative images before (middle) and after 3 min treatment, with or without UV irradiation (bottom) across three conditions of Tetra-motif: (i) w/UV, w/o PC spacer, (ii) w/o UV, w/PC spacer, and (iii) w/UV, w/PC spacer. Scale bars represent 100 μm. More details in Figure S11. (c) Illustration depicting the setup for investigating the physical properties of the condensate after deformation of the double-stranded edges of the DNA tetrahedron monomer. Melting temperature (*T*_m_) of all edges is indicated. (d) Representative image sequence showing morphological changes of condensate over time. Scale bars represent 10 μm. (e) Plot of mean circularity changes of the condensate against elapsed time. Error bars represents ± SD. Details in Figure S12.

Another demonstration of controlling the string-based condensates by external stimuli was temperature responsivity (Figures 6c–6e). We attempted deforming the double-stranded edges of the Tetra-motif by heating the DNA condensate up to 68 °C (Figure 6c), which was higher than the melting temperature (*T*_m_) of three out of six edges of the Tetra-motif and significantly lower than the *T*_m_ of the DNA bridge (ligated sticky ends and L-linker) between Tetra-motifs (88.5 °C). The observation was set up as shown in Figure 6c, and the morphological changes in the condensate were observed (Figure 6d): The amorphous condensate gradually adopted a more rounded morphology over 400 min. The circularity of the condensates was quantified and plotted against the elapsed time (Figure 6e). The circularity of the condensates increased from the beginning until 200 min and stabilized. It is also worth noting that the fluorescence intensity of the condensate increases from the beginning until 200 min, suggesting sphericalization of the condensate. While some degree of dissociation may occur due to partial melting of double-stranded DNA, the overall increase of circularity and fluorescence intensity suggests a thermally induced structural reorganization rather than simple dissipation. This is likely due to the increase of flexibility of the string-like structures after deformation of Tetra-motif edges. This observation demonstrates the thermal responsivity of the string-based condensates and suggests the importance of the string-like structures to form the string-based condensate, even with deformed Tetra-motif structures.

## Conclusion

Anisotropic DNA tetrahedral nanostructures (Tetra-motif) could form rigid string-like structures with specified directions along the string, which were directly observed by AFM. It was demonstrated that a string-based DNA condensate was successfully formed through the physical entanglement of the string-like structures. This string-based condensate was shown to undergo structural rearrangement into a fibrous structure upon stretching and was also demonstrated to be flexible and adaptable to the external environment. The increase in the viscosity of this DNA condensate with an increase in the length of the string-like structures through ligation agrees with the prediction of the reptation model, which further supports the views of the string-based DNA condensate as an entangled polymer system.

The observed scaling relationship of *D* ∝ *L* ^–2.4^ is reasonably close to the *L* ^–2^ prediction of the reptation model. This slight deviation may result from experimental variability in length measurements, heterogeneity in entanglement due to variation of the string length of condensate, or imperfect fluorescence normalization. More DNA condensate samples with varying string-like structures or polymer lengths are required to improve data reliability. Future studies on controlling the length of the string-like structures for a broader range of polymer lengths will improve our understanding of the DNA-condensate system. Furthermore, the “bead-and-string” architecture, where bulky Tetra-motifs alternate with linkers, may limit sliding between string-like structures and potentially contribute to condensate formation. Further study of the tetrahedron-size dependency may provide more information for the formation process.

The entangled string-based DNA condensate offers unique functional advantages. The microfluidic experiments demonstrated the capability of conforming to the external environment, whereas a cross-linked DNA condensate has a hydrogel-like more rigid feature. These results suggest that the string-based condensate is an intermediate between the DNA droplets and DNA hydrogels. Unlike highly dynamic DNA droplets, which are structurally unstable owing to their liquid-like nature, the string-based DNA condensates were more stable than DNA droplets but deformable under mechanical stress. Moreover, the change in morphology in response to UV irradiation and heat (Figure 6) demonstrates stimulus-responsive behavior. This suggests that it has the potential to be designed for specialized functions in which specific stimuli can be introduced to trigger the desired response, implying broad functional applications.

A balance of flexible and stable properties enables penetration and shape conformation of irregular tissue architectures, offering a viable option as a drug delivery vehicle. Furthermore, the incorporation of the DNA tetrahedron, which is highly capable in medical applications^78–84^ as the monomer, further improves the versatility of the entangled string-based DNA condensate. The ability of the condensate made up of DNA tetrahedral polymers to disintegrate into individual monomers that are free for cell penetration in response to UV irradiation further would demonstrate its potential for controlled and dynamic therapeutic applications such as targeted drug delivery and responsive biomaterials.

## Materials and Methods

### Sample Preparation

All DNA strands were synthesized using Eurofins Genomics (Tokyo, Japan), high-performance liquid chromatography grade, except for the PC spacer-inserted DNA, which was provided by Tsukuba Oligo Service (Ibaraki, Japan), high-performance liquid chromatography grade. All DNA strands were stored at −20 °C in 100 μM concentration. All DNA sequences used are given in Table S1, and their melting temperatures are given in Table S2.

Monomers and linkers were assembled by using a combination of DNA strands (Tables S3 and S4) in a buffer solution containing 20 mM Tris-HCl (pH 8.0) and 350 mM NaCl. For tetrahedra, mixtures were incubated at 95 °C for 3 min before cooling down to 4 °C at −5 °C/s using a real-time PCR system (CFX Connect, Bio-Rad, Hercules, CA, USA). For linkers, mixtures were heated to 95 °C for 3 min before cooling down to 75 °C followed by 4 °C at −1 °C/min using a thermal cycler (Mastercycler Nexus X2, Eppendorf, Hamburg, Germany). X-motif was assembled by heating to 95 °C for 3 min and then cooled to 4 °C at a rate of −1 °C/min using a thermal cycler, as previously reported.

Monomers were then combined with their corresponding linker at a 1 μM equimolar final concentration. For the condition with ligation, the mixture containing 1 U/μL T4 DNA Ligase with 1× ligation buffer (catalog no. 2011 B), provided by Takara Bio (Shiga, Japan), was incubated at 16 °C for 24 h. For the condition without ligation, the mixture contained 20 mM Tris-HCl (pH 8.0) and 350 mM NaCl and was incubated at 50 °C for 30 min before storage at 4 °C.

Then, 400 μL of the Monomer–Linker mixture was added into a centrifugal filter (Amicon Ultra 0.5 mL 30 kDa cutoff, UFC503024, Merck Millipore, Darmstadt, Germany) and spun down at 14,000 × *g* for 5 min, followed by washing twice with 400 μL of buffer (20 mM Tris-HCl (pH 8.0) and 350 mM NaCl) with the same condition. The concentrated sample was recovered at 1,000 × *g* for 2 min into a fresh tube and stored at 4 °C. The volume after concentration is estimated to be 50 μL, which is a 4 μM concentration of DNA monomer. The concentrated samples were allowed to sit for 14 days at 4 °C to maximize the pellet sediment available for the experiment.

### Confocal Microscopy Observation

FAM-labeled DNA strands were added to the mixture for monomer assembly at 1/10 the concentration of the corresponding one out of four main component DNA strands, as indicated in (Table S3), as previously reported.^27^ The assembly was visualized with an excitation wavelength of 473 nm by using a confocal laser scanning microscope (FV1000, Olympus, Tokyo, Japan). A glass slide measuring 30 × 40 mm with a thickness of 0.17 mm (no.1, Matsunami Glass Ind., Ltd., Kishiwada, Japan). The glass slides were coated with bovine serum albumin (BSA) by treatment with a solution of 5% (w/v) BSA (FUJIFILM Wako Pure Chemical Corp.) and 20 mM Tris–HCl before drying under an airflow. A silicone sheet (1.0 mm) was punched with a hole and cut in size before being laid onto a BSA-coated glass slide as a bank. The sample was pipetted onto a glass slide and covered with mineral oil (Nacalai Tesque, Kyoto, Japan). The glass slide with the sample was placed on a stage heater (Model 10021 (Linkam PE-120 with a T96-P controller); Japan High Tech Co., Fukuoka, Japan). The observation temperature was set at 30 °C unless mentioned otherwise. For the FRAP experiment, DNA condensates were bleached at 405 nm, 60% intensity for 30 s, and a bleaching circle of 6 μm diameter.

### Gel Electrophoresis

Native PAGE was conducted with 6% polyacrylamide gel prepared by mixing Tris-acetate-EDTA buffer (Nacalai Tesque), acrylamide/Bis (19:1) solution (Nacalai Tesque), ammonium peroxodisulfate (FUJIFILM Wako Pure Chemical, Osaka, Japan), and N,N,N′,N′-tetramethylethylenediamine (FUJIFILM Wako Pure Chemical). DNA samples were mixed with a loading buffer containing 0.05% (w/v) bromophenol blue, 10% (v/v) glycerol (FUJIFILM Wako Pure Chemical, Osaka, Japan), and 10 mM EDTA (pH 8.0) (Nippon Gene, Toyama, Japan) before running at 200 V in 1× Tris-acetate-EDTA buffer at room temperature.

Denaturing PAGE was conducted with 10% denaturing polyacrylamide gel containing 8 M urea (FUJIFILM Wako Pure Chemical), acrylamide/Bis (29:1) solution (Nacarai Tesque), Tris-borate EDTA buffer (Nippon Gene), ammonium peroxodisulfate (FUJIFILM Wako Pure Chemical), and N,N,N′,N′-tetramethylethylenediamine (FUJIFILM Wako Pure Chemical). DNA samples were mixed with loading buffer containing 0.24% (w/v) bromophenol blue, 12 mM EDTA (pH 8.0) (Nippon Gene), and ∼ 100% (v/v) formamide (FUJIFILM Wako Pure Chemical) followed by incubation at 95 °C for 10 min. Electrophoresis was performed at 200 V in 1× Tris-borate EDTA buffer at room temperature.

### AFM Observation

The DNA condensate (pellet) was diluted 20×, pipetted, and spin-coated onto freshly cleaved mica at 3000 rpm for 30 s on an Opticoat SpinCoater (Mikasa Co., Ltd., Tokyo, Japan). It was then covered with 20 μL of 4 mM NiCl for 30 min before observing using Bruker Nanoscope V multimode 8 (Bruker Corp., Massachusetts, U.S.A.) with a SCANASYST Fluid+ cantilever probe.

### Microfluidic Device Experiment

The microfluidic device was fabricated by the standard PDMS replica molding, followed by the hydrophilic treatment of channel walls by the block copolymer surfactant (1%v/v Pluronic F68, Sigma-Aldrich), as previously reported.^95^ First, 1 μL of 10× diluted DNA condensate was pipetted onto the dropping section and allowed to flow through the hydrophilized channel. Observations were conducted under an IX81 inverted compound microscope (Olympus Co., Tokyo, Japan) attached to a CSU-X1 confocal scanning unit (Yokogawa Electric Co., Tokyo, Japan) and an iXon X3 EMCCD camera (Oxford Instruments Andor Ltd., Belfast, U.K.) irradiated with a 473 nm laser. For quantitative analysis, only particles of condensate larger than 10 μm in area are considered, assuming a spherical shape. Representative data are provided (Movies S7, S8, and S9), and the size-frequency distribution is plotted in Figure S3.

### UV-Responsive DNA Condensate Experiments

The DNA condensate was irradiated with a focused UV light beam at 365 nm from a MAX-303 xenon light source paired with a KLQ-2.5 lens and a corresponding filter (Asahi Spectra Co., Ltd., Tokyo, Japan) at maximum intensity for 3 min.

## Supplementary Material





















## Notes

Oligonucleotide sequences; sequence section melting points; monomer and linker DNA composition; statistical analysis; confocal microscopy images; full gel images of polyacrylamide gel electrophoresis; size-frequency distribution of condensate pass-through and trapped; FRAP experimental methodology; AFM images; circularity curves of each sample; and plot of recovered intensity after bleaching against length of condensate (DOCX)
Movie S1 of DNA condensate manipulated by a pipet tip (MP4)
Movie S2 of DNA condensate manipulated by a pipet tip (MP4)
Movie S3 of DNA condensate manipulated by a pipet tip (MP4)
Movie S4 of motion of DNA condensate particles in the microfluidic channel (MP4)
Movie S5 of motion of DNA condensate particles in the microfluidic channel (MP4)
Movie S6 of motion of DNA condensate particles in the microfluidic channel (MP4)
Movie S7 of motion of DNA condensate particles in the microfluidic channel (MP4)
Movie S8 of motion of DNA condensate particles in the microfluidic channel (MP4)
Movie S9 of motion of DNA condensate particles in the microfluidic channel (MP4)
