# Supplementary material for: DNA Condensates via Entanglement of String-like Structures Based on Anisotropic Nanotetrahedra
Source: JACS Au. 2025 Jun 10;5(7):3249–61. doi: 10.1021/jacsau.5c00421 (PMC12308406; doi:10.1021/jacsau.5c00421)
Supplement: Supplementary file 1 [file au5c00421_si_001.docx]

Supplementary Information

DNA condensates via entanglement of string-like structures based on anisotropic nano-tetrahedra

Hong Xuan Chai^1^, Kanta Kayanuma^2^, Hiroaki Suzuki^2^, Masahiro Takinoue^1, 3, 4,*^

^1^Department of Life Science and Technology, Institute of Science Tokyo, Kanagawa 226-8501, Japan

^2^Department of Precision Mechanics, Graduate School of Science and Engineering, Chuo University, 1-13-27 Kasuga, Bunkyo-ku, Tokyo 112-8551, Japan

^3^Department of Computer Science, Institute of Science Tokyo, Kanagawa 226-8501, Japan

^4^Research Center for Autonomous Systems Materialogy (ASMat), Institute of Integrated Research, Institute of Science Tokyo, Kanagawa 226-8501, Japan

*Corresponding author. E-mail address: takinoue@comp.isct.ac.jp

**Contents:**

**Oligonucleotide sequences**

**Materials composition**

**Statistical analysis**

**Supplementary figures**

**Captions for supplementary movies**

**Oligonucleotide sequences**

**Table S1:** Oligonucleotide sequences.

| Name | Sequence (5' - 3') |
| --- | --- |
| Tetra1_LongSE | [PHO]**CCGTAGCTGTTGATCGTCAAA**TTTCAACTGCCTGGTGATAAAACGACACTACGTGGGAATCTACTATGGCGGCTCTTC |
| Tetra2_LongSE | [PHO]**CCGTAGCTGTTGATCGTCAAA**TTTTCAGACTTAGGAATGTGCTTCCCACGTAGTGTCGTTTGTATTGGACCCTCGCAT |
| Tetra3_ShortSE | GAATTGGAGACATTACATTCCTAAGTCTGAAACATTACAGCTTGCTACACGAGAAGAGCCGCCATAGTA |
| Tetra4_ShortSE | GAATTGGAGACATTTATCACCAGGCAGTTGACAGTGTAGCAAGCTGTAATAGATGCGAGGGTCCAATAC |
| Tetra3_ShortSE_3FAM | GAATTGGAGACATTACATTCCTAAGTCTGAAACATTACAGCTTGCTACACGAGAAGAGCCGCCATAGTA[FAM] |
| Tetra1_noLongSE | TCAACTGCCTGGTGATAAAACGACACTACGTGGGAATCTACTATGGCGGCTCTTC |
| Tetra2_noLongSE | TTCAGACTTAGGAATGTGCTTCCCACGTAGTGTCGTTTGTATTGGACCCTCGCAT |
| Tetra3_noShortSE | ACATTCCTAAGTCTGAAACATTACAGCTTGCTACACGAGAAGAGCCGCCATAGTA |
| Tetra4_noShortSE | TATCACCAGGCAGTTGACAGTGTAGCAAGCTGTAATAGATGCGAGGGTCCAATAC |
| Tetra3_noShortSE_3FAM | ACATTCCTAAGTCTGAAACATTACAGCTTGCTACACGAGAAGAGCCGCCATAGTA[FAM] |
| LL1 | **TTTGACGATCAACAGCTACGG**ACGGAGACGGTCTGTTATTGA |
| LL2 | **TTTGACGATCAACAGCTACGG**TCAATAACAGACCGTCTCCGT |
| SL1 | TGTCTCCAATTCTCACACACCTATTTACTCCCT |
| SL2 | TGTCTCCAATTCAGGGAGTAAATAGGTGTGTGA |
| X1_ShortSE | GAATTGGAGACACTGGACTAACGGAACGGTTAGTCAGGTATGCCAGCA |
| X2_LongSE | [PHO]**CCGTAGCTGTTGATCGTCAAA**TGCTGGCATACCTGACTTTCGCAAATTTACAGCGCC |
| X3_ShortSE | GAATTGGAGACAGGCGCTGTAAATTTGCGTTCATCACTTGGGACCATG |
| X4_LongSE | [PHO]**CCGTAGCTGTTGATCGTCAAA**CATGGTCCCAAGTGATGTTCCGTTCCGTTAGTCCAG |
| X1_ShortSE_3FAM | GAATTGGAGACACTGGACTAACGGAACGGTTAGTCAGGTATGCCAGCA[FAM] |
| LL1_PCS | **TTTGACGATCAACAGCTACGG**ACGGAGACGG[PC spacer]TCTGTTATTGA |
| LL2_PCS | **TTTGACGATCAACAGCTACGG**TCAATAACAGA[PC spacer]CCGTCTCCGT |

**Table S2:** Melting temperature, T_m_ of sequence pairs determined using NUPACK with 350 mM Na^+^ and DNA strands at 1µM each with ‘dna04’ parameter and ensemble set as ‘All stacking’.

| Sequence pair | T_m_ [°C] |
| --- | --- |
| ACATTCCTAAGTCTGAA vs TTCAGACTTAGGAATGT | 58.5°C |
| ATTACAGCTTGCTACAC vs GTGTAGCAAGCTGTAAT | 61.0°C |
| GAAGAGCCGCCATAGTA vs TACTATGGCGGCTCTTC | 71.0°C |
| TATCACCAGGCAGTTGA vs TCAACTGCCTGGTGATA | 68.0°C |
| ATGCGAGGGTCCAATAC vs GTATTGGACCCTCGCAT | 68.5°C |
| ACGACACTACGTGGGAA vs TTCCCACGTAGTGTCGT | 65.5°C |
| **CCGTAGCTGTTGATCGTCAAA** vs **TTTGACGATCAACAGCTACGG** | 69.5°C |
| GAATTGGAGACA vs TGTCTCCAATTC | 42.0°C |
| ACGGAGACGGTCTGTTATTGA vs TCAATAACAGACCGTCTCCGT | 69.5°C |
| TCACACACCTATTTACTCCCT vs AGGGAGTAAATAGGTGTGTGA | 69.0°C |
| CTGGACTAACGGAACGG vs CCGTTCCGTTAGTCCAG | 64.5°C |
| AGTCAGGTATGCCAGCA vs TGCTGGCATACCTGACT | 67.5°C |
| CGCAAATTTACAGCGCC vs GGCGCTGTAAATTTGCG | 65.5°C |
| CATCACTTGGGACCATG vs CATGGTCCCAAGTGATG | 65.5°C |

**Materials composition**

**Table S3:** DNA strand concentration for different monomer assemblies.

| Monomer | Name of DNA stand | Final concentration |
| --- | --- | --- |
| Tetra-motif | Tetra1_LongSE | 1.18 |
|  | Tetra2_LongSE | 1.18 |
|  | Tetra3_ShortSE | 1.062 |
|  | Tetra3_ShortSE_3FAM | 0.118 |
|  | Tetra4_ShortSE | 1.18 |
| Tetra-motif_noShortSE | Tetra1_LongSE | 1.18 |
|  | Tetra2_LongSE | 1.18 |
|  | Tetra3_**no**ShortSE | 1.062 |
|  | Tetra3_**no**ShortSE_3FAM | 0.118 |
|  | Tetra4_**no**ShortSE | 1.18 |
| Tetra-motif_noSE | Tetra1_**no**LongSE | 1.18 |
|  | Tetra2_**no**LongSE | 1.18 |
|  | Tetra3_**no**ShortSE | 1.062 |
|  | Tetra3_**no**ShortSE_3FAM | 0.118 |
|  | Tetra4_**no**ShortSE | 1.18 |
| X-motif | X1_ShortSE | 1.062 |
|  | X2_LongSE | 1.18 |
|  | X3_ShortSE | 1.18 |
|  | X4_LongSE | 1.18 |
|  | X1_ShortSE_3FAM | 0.118 |

Table S4: DNA strand concentration for S-linker, L-linker and S-linker + L-linker.

| Linker | Components | Final concentration |
| --- | --- | --- |
| S-linker | SL1 | 1.18 |
|  | SL2 | 1.18 |
| L-linker | LL1 | 1.18 |
|  | LL2 | 1.18 |
| S-linker + L-linker | SL1 | 1.18 |
|  | SL2 | 1.18 |
|  | LL1 | 1.18 |
|  | LL2 | 1.18 |
| PC spacer-inserted | LL1_PCS | 1.18 |
| L-linker | LL2_PCS | 1.18 |

**Statistical analysis**

**Table S5:** P-value of paired wise comparison of conditions in Figure S5 determined using two-tailed Welch’s t-test.

| Length (nm) | | | Length (unit monomer) | | |
| --- | --- | --- | --- | --- | --- |
| Group 1 | Group 2 | P-value | Group 1 | Group 2 | P-value |
| i | ii | 7.83E-09 | i | ii | 1.19.E-03 |
| i | iii | 1.65E-15 | i | iii | 1.64.E-14 |
| ii | iii | 1.10E-11 | ii | iii | 1.10.E-11 |
| iv | v | 5.80E-11 | iv | v | 5.78E-10 |
| iv | vi | 2.92E-11 | iv | vi | 1.53E-10 |
| v | vi | 7.62E-03 | v | vi | 7.62.E-03 |
| i | iv | 2.38E-01 | i | iv | 2.38.E-01 |
| ii | v | 1.36E-06 | ii | v | 1.36.E-06 |
| iii | vi | 7.80E-01 | iii | vi | 7.80.E-01 |
| vii | viii | 1.52E-04 |  |  |  |
| vii | ix | 2.34E-09 |  |  |  |
| viii | ix | 5.06E-02 |  |  |  |

**Supplementary figures**


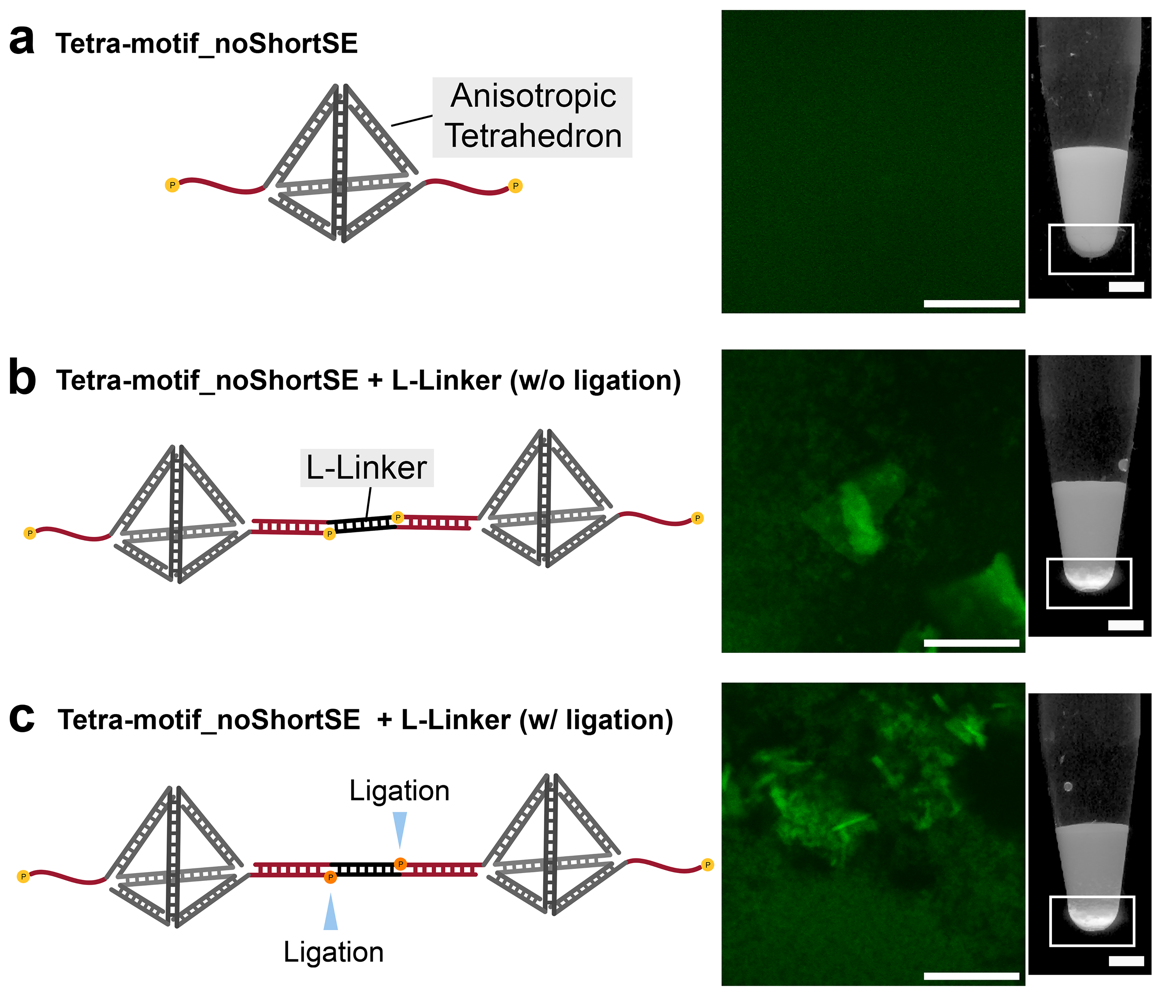


**Figure S1:** Illustration of Tetra-motif without short sticky ends under different condition (left), corresponding CLSM images (middle), and pictures of pellet in a test tube (right): (a) Tetra-motif_noShortSE, (ii) Tetra-motif_noShortSE + L-linker (w/o ligation), (iii) Tetra-motif_noShortSE + L-linker (w/ ligation). Scale bars: 50 µm (CLSM images), 2 mm (pellet pictures).


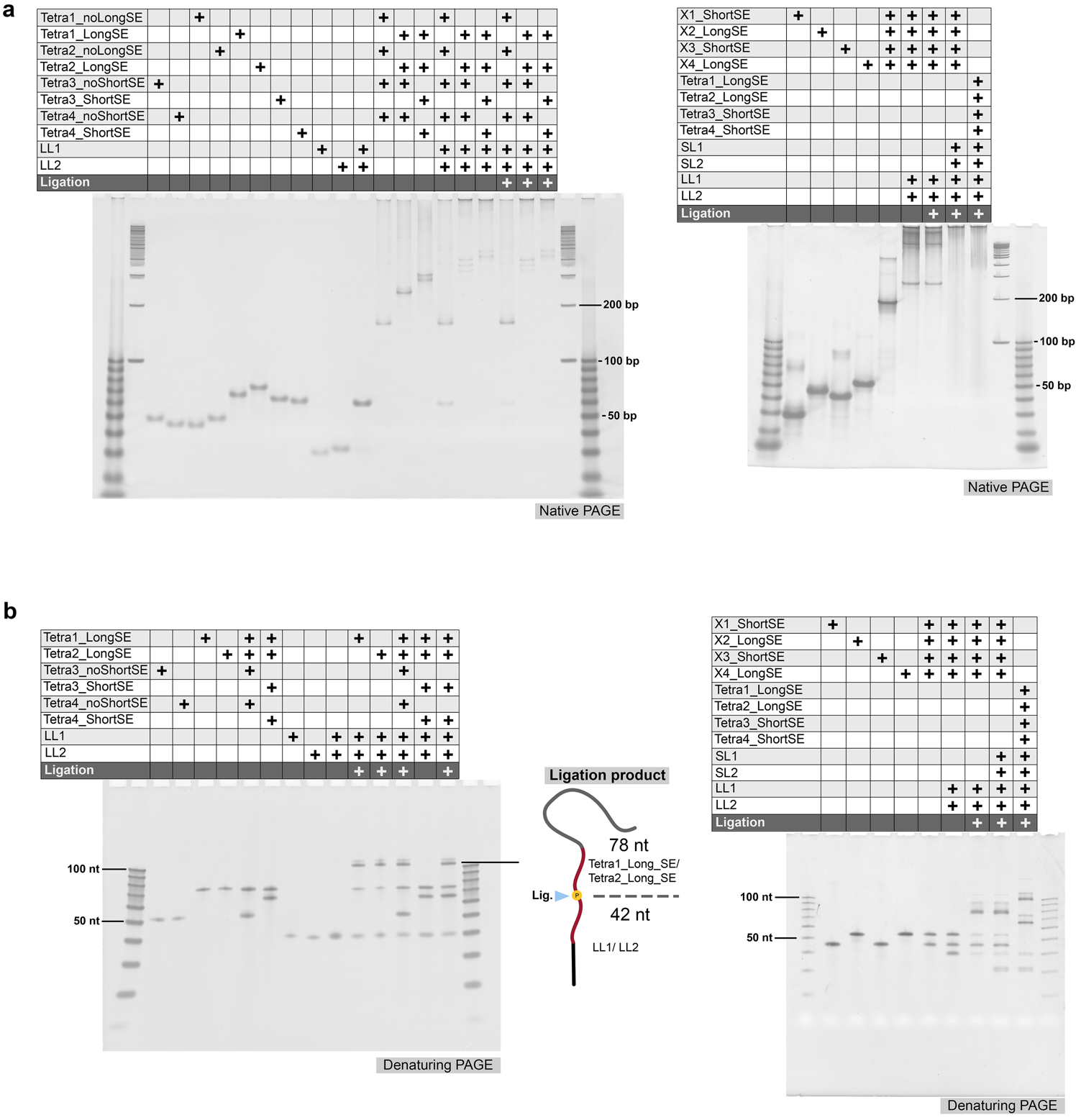


**Figure S2:** Polyacrylamide gel electrophoresis (PAGE) for verification of DNA tetrahedron and X-motif assemblies and structure formation. (a) Native PAGE confirming the presence of monomer assemblies and the formation of low mobility structure. (b) Denaturing PAGE verifying the presence of the desired ligation products.


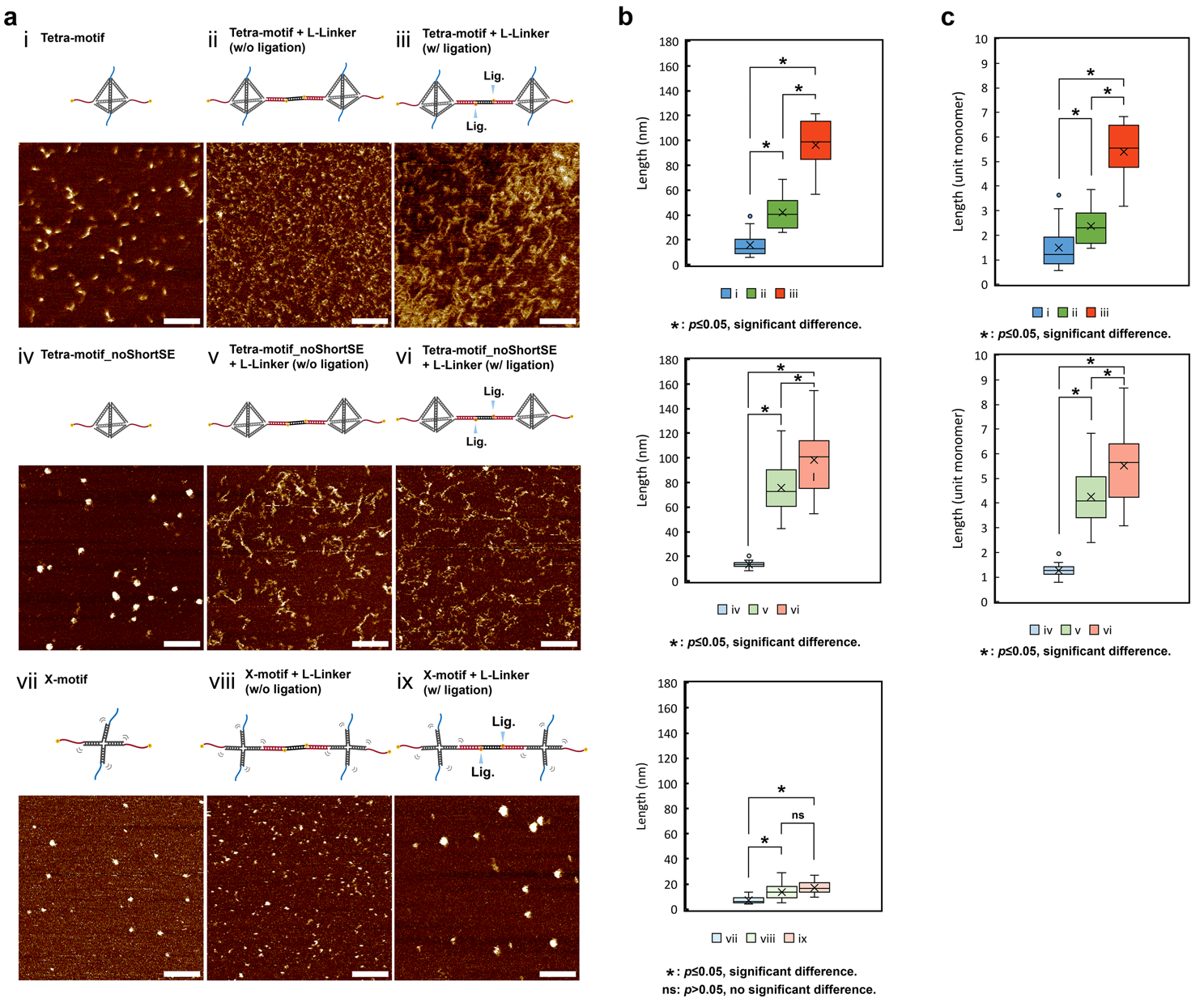


**Figure S3:** Atomic Force Microscopy (AFM) analysis of string-like structures composing condensates. (a) Illustration and representative images of three conditions of three different monomers. Scale bars represent 100 nm. (b) Box plot of mean string lengths in nanometers (nm), corresponding to each condition (i) through (ix) as shown in (a). (c) Box plot of mean string lengths in unit monomer estimated, corresponding to each condition (i) through (ix) as shown in (a). Length per unit Tetra-motif is estimated to be 10.7 nm based on measurement. Length per unit Tetra-motif with the presence of L-linker is estimated to be 17.9 nm, with 1 bp = 0.34 nm. All box plots indicate the median (center line), interquartile range (box), and 1.5× IQR (whiskers). The “X” marks the group mean. Statistical comparisons were performed using two-tailed Welch’s t-tests. Significance is indicated as *p* ≤ 0.05 (*), and *p* > 0.05 (ns). Scale bars represent 100 nm.


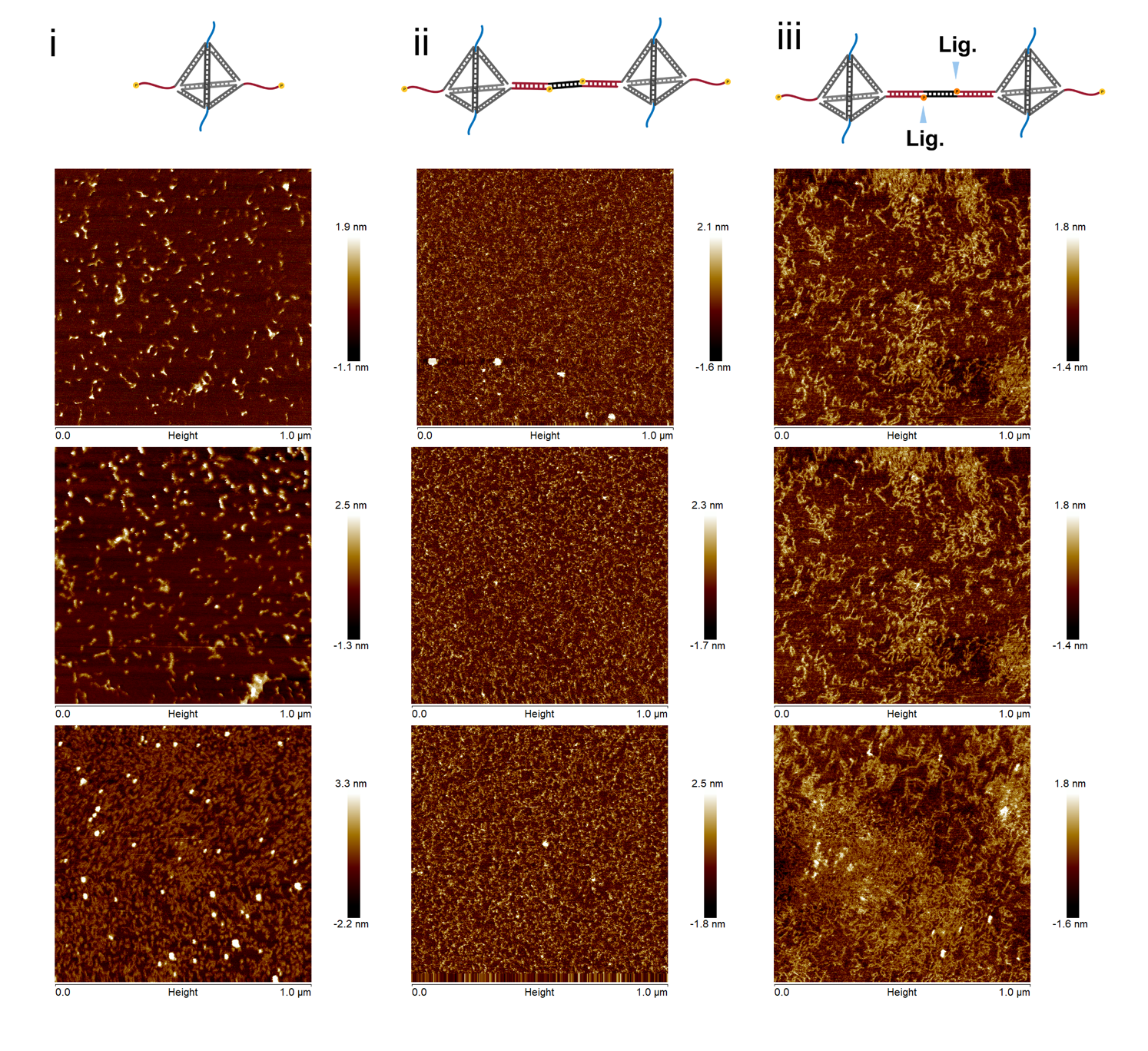


**Figure S4:** Uncropped Atomic Force Microscopy (AFM) images which 20 random measurement was conducted for statistical analysis. (i) Tetra-motifs only, (ii) Tetra-motifs + L-linkers (w/o ligation), (iii) Tetra-motifs + L-linkers (w/ ligation).


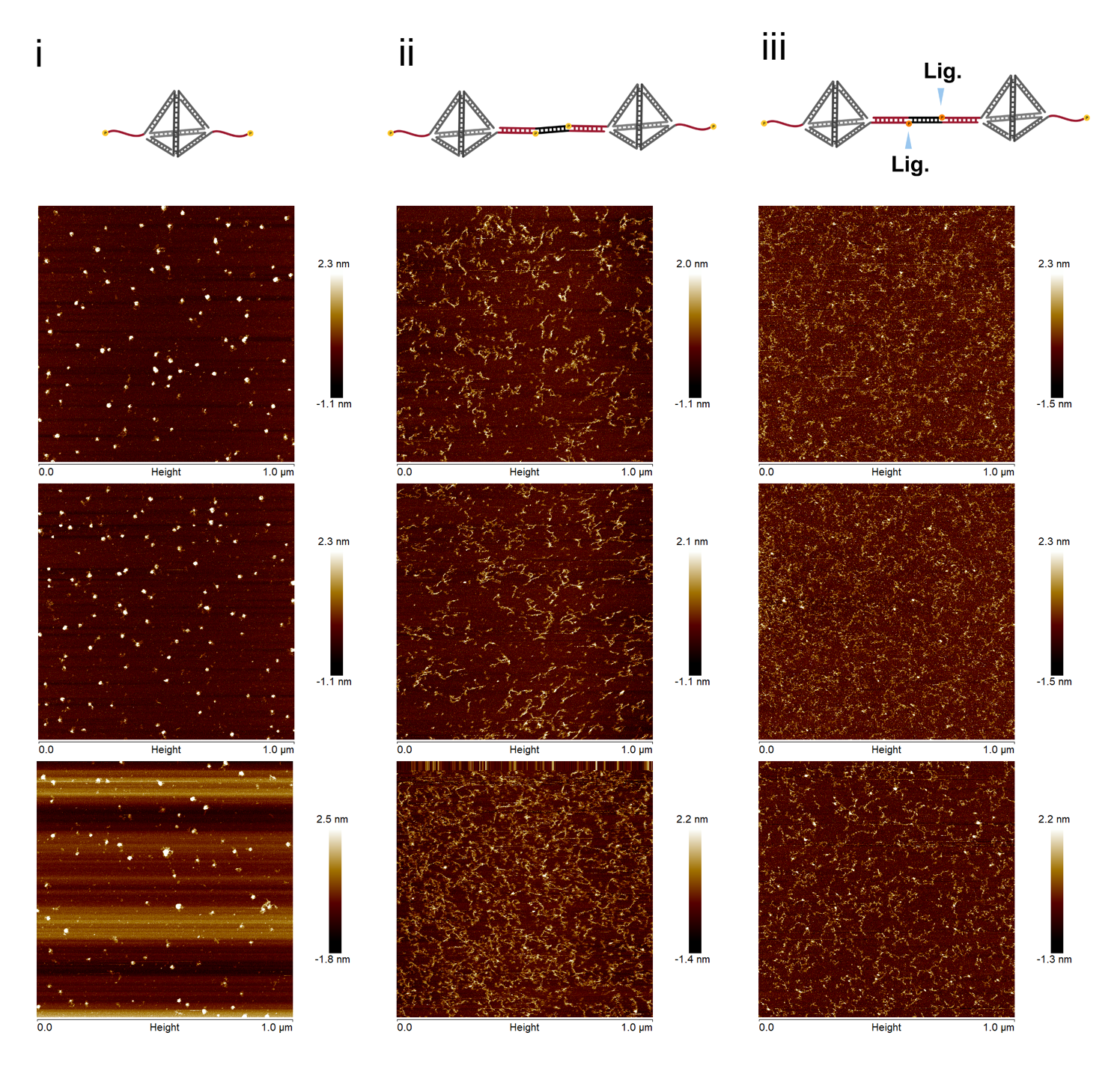


**Figure S5:** Uncropped Atomic Force Microscopy (AFM) images which 20 random measurement was conducted for statistical analysis. (i) Tetra-motif_noShortSEs only, (ii) Tetra-motif_noShortSEs + L-linkers (w/o ligation), (iii) Tetra-motif_noShortSEs + L-linkers (w/ ligation).


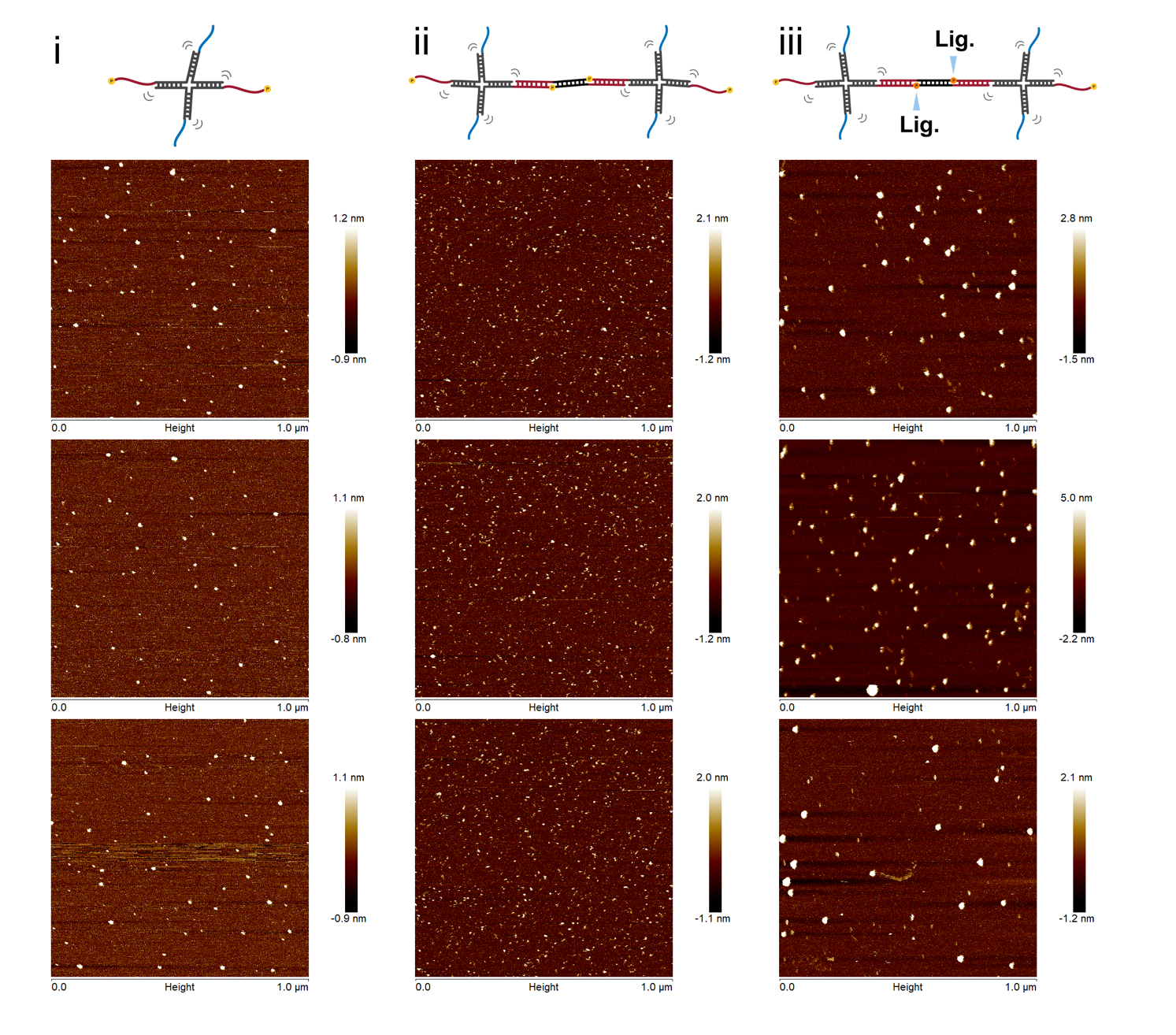


**Figure S6:** Uncropped Atomic Force Microscopy (AFM) images which 20 random measurement was conducted for statistical analysis. (i) X-motifs only, (ii) X-motifs + L-linkers (w/o ligation), (iii) X-motifs + L-linkers (w/ ligation).


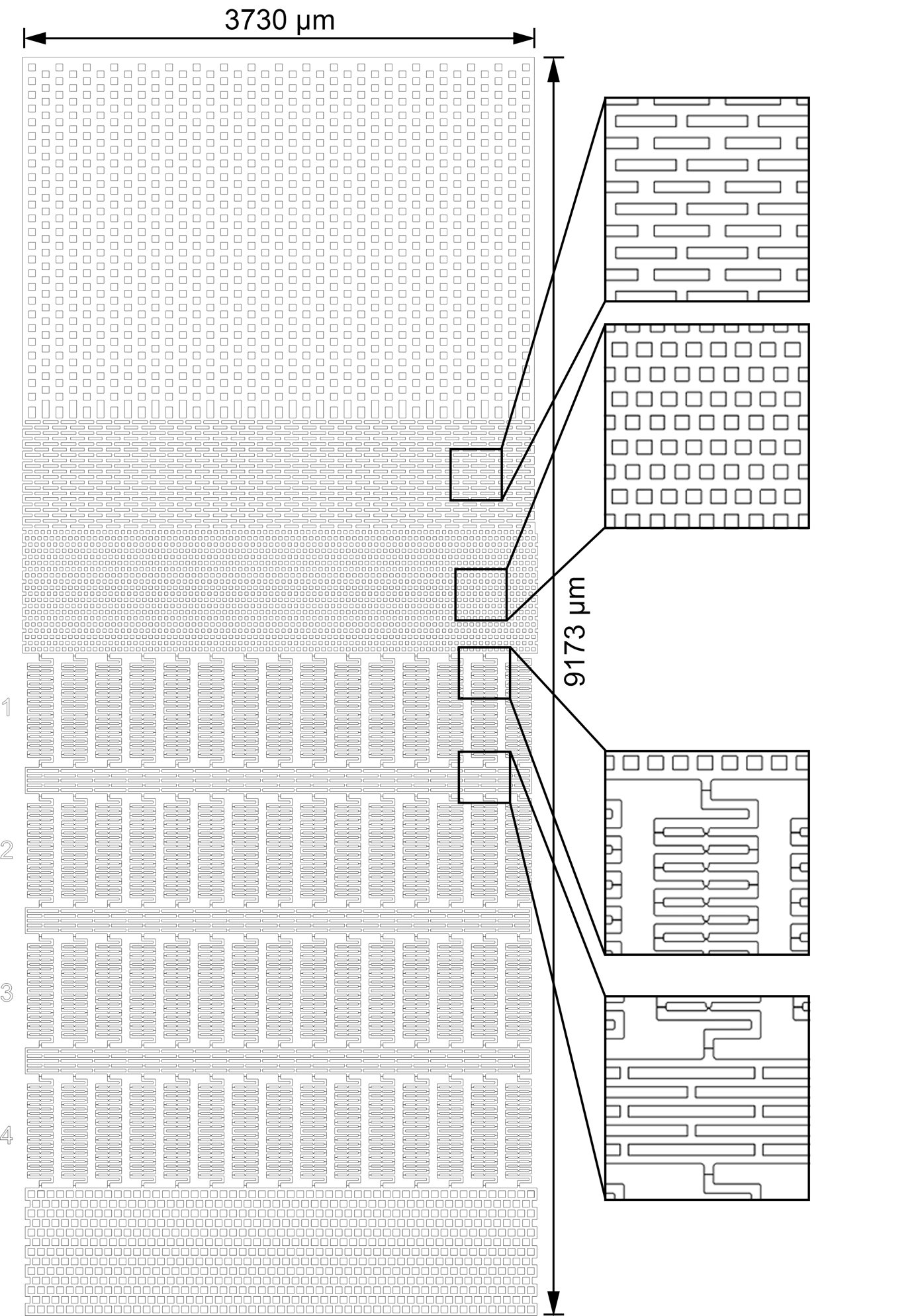


**Figure S7:** High-resolution schematic of the microfluidic trap device mentioned in Figure 4.


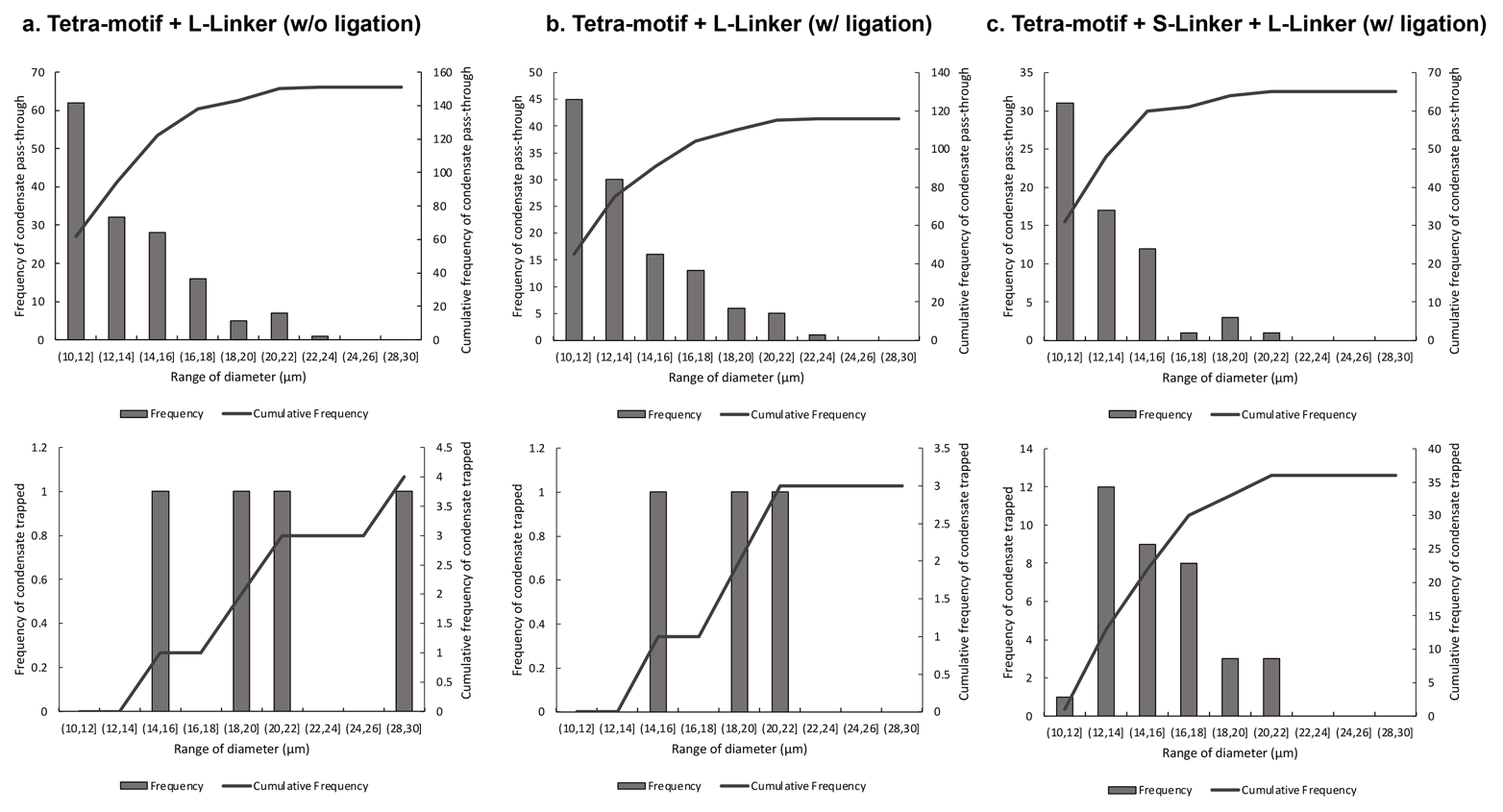


**Figure S8:** Size-frequency distribution of condensate particle pass-through (top) and condensate particle trapped (bottom) corresponding to Figure 4.


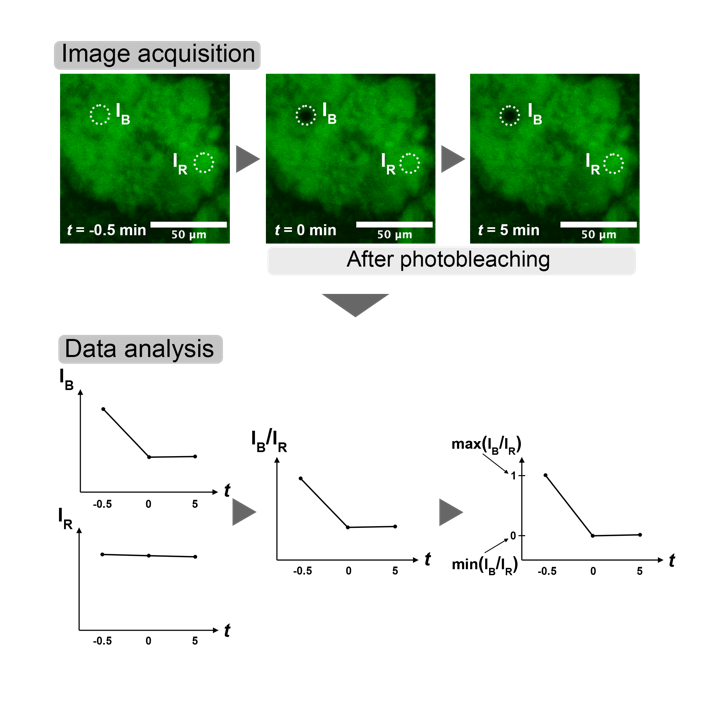


**Figure S9:** Depiction of method of FRAP experiment in Figure 5.


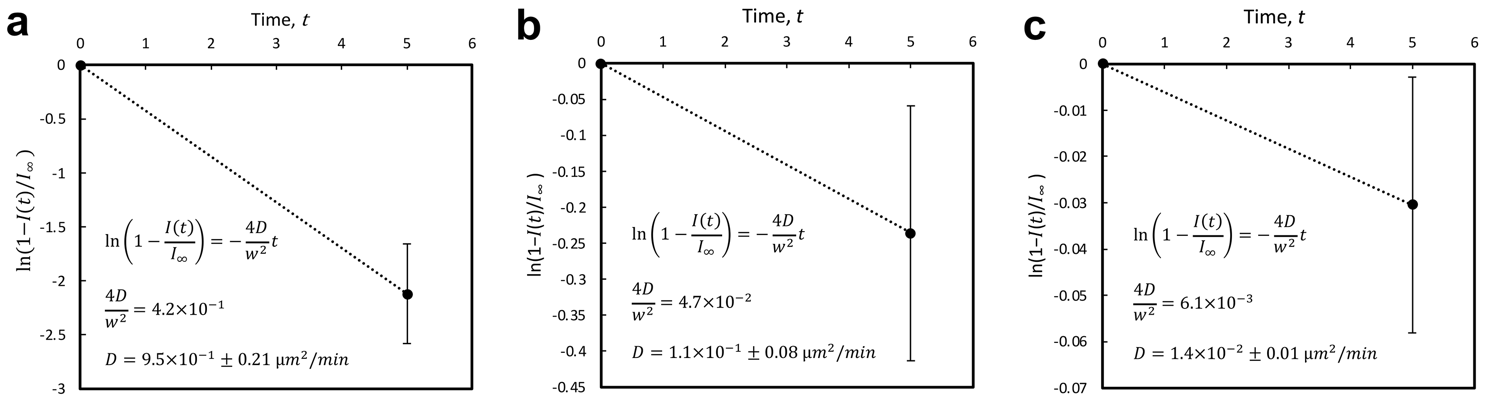


**Figure S10:** Estimation of the diffusion coefficient $D$ from the linearized FRAP recovery model for three different conditions. (a) Tetra-motif, (b) Tetra-motif + L-linker (w/o ligation), and (c) Tetra-motif + L-linker (w/ ligation). Plots of $ln\left( 1-I(t)/I_{\infty} \right)$versus time $t$ were shown for each condition. Fluorescence intensity $I(t)$ was normalized to the pre-bleach intensity, and full recovery was assumed $I_{\infty}\approx I_{\mathrm{pre}}$. The dotted lines represent linear fits to the FRAP model $ln\left( 1-I(t)/I_{\infty} \right)=-(4D/w^{2})\cdot t$. The slope of each fit was used to calculate the diffusion coefficient $D$, with the uncertainty estimated by propagating the standard deviation of the normalized fluorescence intensity $I(t=5 min)$. Numerical values of $4D/w^{2}$and the corresponding $D$ are shown within each panel.


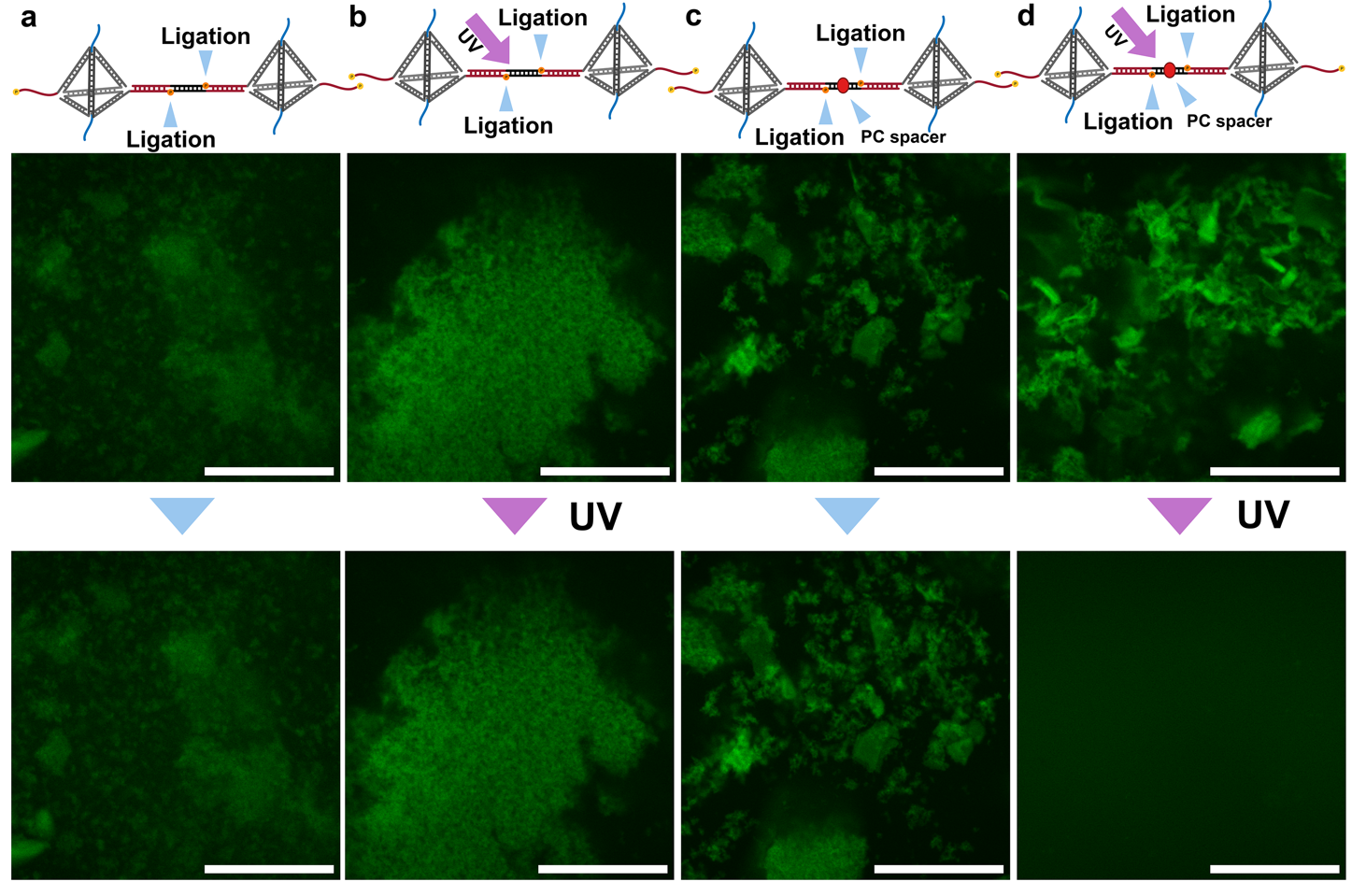


**Figure S11:** Investigating disintegration of DNA condensate formed by string-like structure connected via photocleavable (PC) spacer-inserted L-linker, following ultraviolet (UV) irradiation with illustration (top) and representative images before (middle) and after 3 min treatment, with or without UV irradiation (bottom) across four conditions of Tetra-motif: (a) w/o UV, w/o PC spacer, (b) w/ UV, w/o PC spacer, (c) w/o UV, w/ PC spacer, and (d) w/ UV, w/ PC spacer. Scale bars represent 100 µm.


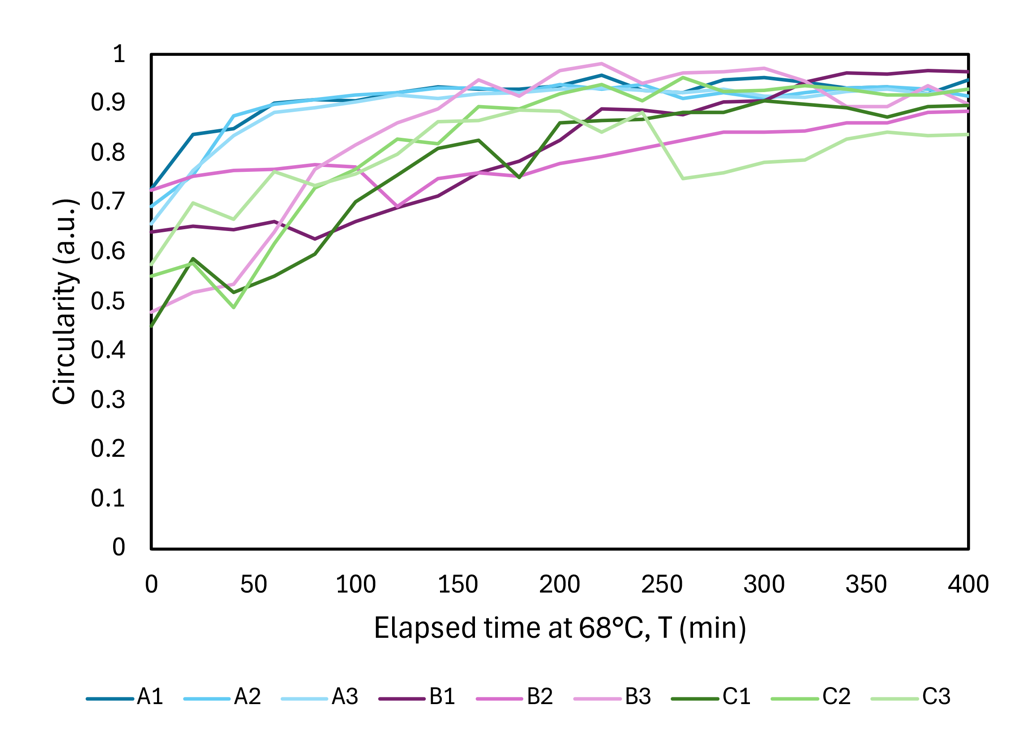


**Figure S12:** Circularity curves of triplicate of three samples corresponding to Figure 6e. $Circularity=4\Pi\cdot{Area}/{{Perimeter}^{2}}$

**Captions for supplementary movies**

**Movie S1:** DNA condensate, Tetra-motif + L-linker (w/o ligation) manipulated by a pipette tip as observed in a standard 0.2 mL PCR tube.

**Movie S2:** DNA condensate, Tetra-motif + L-Linker (w/ ligation) manipulated by a pipette tip as observed in a standard 0.2 mL PCR tube.

**Movie S3:** DNA condensate, Tetra-motif + S-Linker + L-Linker (w/o ligation) manipulated by a pipette tip as observed in a standard 0.2 mL PCR tube.

**Movie S4:** Motion of particles of DNA condensate, Tetra-motif + L-Linker (w/o ligation) in microfluidic trap device channel captured at the end of the flow. Scale bar represents 50 µm.

**Movie S5:** Motion of particles of DNA condensate, Tetra-motif + L-Linker (w/ ligation) in microfluidic trap device channel captured at the end of the flow. Scale bar represents 50 µm.

**Movie S6:** Motion of particles of DNA condensate, Tetra-motif + S-Linker + L-Linker (w/o ligation) in microfluidic trap device channel captured at the end of the flow. Scale bar represents 50 µm.

**Movie S7:** Representative data showing the flow of particles of DNA condensate, Tetra-motif+ L-Linker (w/o ligation) in microfluidic trap device channel used for quantitative analysis in Figure 3bi. Scale bar represents 100 µm.

**Movie S8:** Representative data showing the flow of particles of DNA condensate, Tetra-motif + L-Linker (w/ ligation) in microfluidic trap device channel used for quantitative analysis in Figure 3bii. Scale bar represents 100 µm.

**Movie S9:** Representative data showing the flow of particles of DNA condensate, Tetra-motif + S-Linker + L-Linker (w/ ligation) in microfluidic trap device channel used for quantitative analysis in Figure 3biii. Scale bar represents 100 µm.
